# Measuring multiple parameters of CD8+ tumor-infiltrating lymphocytes in human cancers by image analysis

**DOI:** 10.1186/s40425-018-0326-x

**Published:** 2018-03-06

**Authors:** Keith E. Steele, Tze Heng Tan, René Korn, Karma Dacosta, Charles Brown, Michael Kuziora, Johannes Zimmermann, Brian Laffin, Moritz Widmaier, Lorenz Rognoni, Ruben Cardenes, Katrin Schneider, Anmarie Boutrin, Philip Martin, Jiping Zha, Tobias Wiestler

**Affiliations:** 1 MedImmune, One MedImmune Way, Gaithersburg, MD 20878 USA; 2 Professional Services, Definiens AG, Bernhard-Wicki-Strasse 5, 80636 Munich, Germany; 3 Present address: Brian Laffin-BMS US Medical Oncology, 3401 Princeton Pike, Lawrence Township, NJ 08648 USA; 4 Present address: Jiping Zha - NGM Biopharmaceuticals, 333 Oyster Point Boulevard, South San Francisco, CA 94080 USA

**Keywords:** Cancer immunotherapy, Tumor microenvironment, CD8, Image analysis, Immuno-oncology, Tumor-infiltrating lymphocytes

## Abstract

**Background:**

Immuno-oncology and cancer immunotherapies are areas of intense research. The numbers and locations of CD8+ tumor-infiltrating lymphocytes (TILs) are important measures of the immune response to cancer with prognostic, pharmacodynamic, and predictive potential. We describe the development, validation, and application of advanced image analysis methods to characterize multiple immunohistochemistry-derived CD8 parameters in clinical and nonclinical tumor tissues.

**Methods:**

Commercial resection tumors from nine cancer types, and paired screening/on-drug biopsies of non–small-cell lung carcinoma (NSCLC) patients enrolled in a phase 1/2 clinical trial investigating the PD-L1 antibody therapy durvalumab (NCT01693562), were immunostained for CD8. Additional NCT01693562 samples were immunostained with a CD8/PD-L1 dual immunohistochemistry assay. Whole-slide scanning was performed, tumor regions were annotated by a pathologist, and images were analyzed with customized algorithms using Definiens Developer XD software. Validation of image analysis data used cell-by-cell comparison to pathologist scoring across a range of CD8+ TIL densities of all nine cancers, relying primarily on 95% confidence in having at least moderate agreement regarding Lin concordance correlation coefficient (CCC = 0.88–0.99, CCC_lower = 0.65–0.96).

**Results:**

We found substantial variability in CD8+ TILs between individual patients and across the nine types of human cancer. Diffuse large B-cell lymphoma had several-fold more CD8+ TILs than some other cancers. TIL densities were significantly higher in the invasive margin versus tumor center for carcinomas of head and neck, kidney and pancreas, and NSCLC; the reverse was true only for prostate cancer. In paired patient biopsies, there were significantly increased CD8+ TILs 6 weeks after onset of durvalumab therapy (mean of 365 cells/mm^2^ over baseline; *P* = 0.009), consistent with immune activation. Image analysis accurately enumerated CD8+ TILs in PD-L1+ regions of lung tumors using the dual assay and also measured elongate CD8+ lymphocytes which constituted a fraction of overall TILs.

**Conclusions:**

Validated image analysis accurately enumerates CD8+ TILs, permitting comparisons of CD8 parameters among tumor regions, individual patients, and cancer types. It also enables the more complex digital solutions needed to better understand cancer immunity, like analysis of multiplex immunohistochemistry and spatial evaluation of the various components comprising the tumor microenvironment.

**Trial registration:**

ClinicalTrials.gov identifier: NCT01693562.

Study code: CD-ON-MEDI4736–1108.

Interventional study (ongoing but not currently recruiting).

Actual study start date: August 29, 2012.

Primary completion date: June 23, 2017 (final data collection date for primary outcome measure).

**Electronic supplementary material:**

The online version of this article (10.1186/s40425-018-0326-x) contains supplementary material, which is available to authorized users.

## Background

The current high interest in immuno-oncology is due in large part to the recent therapeutic success of approved immunotherapies targeting the programmed cell death protein 1 (PD1) and PD1 ligand (PD-L1) and the cytotoxic T lymphocyte–associated protein 4 (CTLA-4) pathways. Six such drugs are now approved for the treatment of multiple types of cancer. These include the monoclonal antibody therapies durvalumab, nivolumab, avelumab, pembrolizumab, and atezolizumab, which target PD1 and PD-L1 signaling, and ipilimumab, which is directed at CTLA-4 [1–3]. Nonetheless, treatment with these drugs fails in a substantial proportion of patients. There is thus a clear need to investigate the reasons for therapeutic failure, develop biomarkers to identify subsets of patients who are likely to respond, and explore additional aspects of the immune response to cancer.

The immune response to cancer is complex, and our understanding overall seems limited. For example, it is understood that the tumor microenvironment (TME) involves a mix of cell types and cellular and soluble molecules interacting in time and space [3–7]. Understanding how these factors interact spatially represents a particular challenge for the field of immuno-oncology. The Cancer Genome Atlas (TCGA) has substantially informed our understanding of cancer immunity through the mapping of key genomic changes for 33 types of cancer [8]. Importantly, genomic techniques afford comparability across cancer types because of their ability to measure numerous immuno-oncology–related genes simultaneously and because multiple laboratories working independently can coordinate and harmonize their analyses. These kinds of studies, however, cannot be used to measure expressed proteins, the distribution of immune cells in the TME or the spatial arrangement of different cell types.

In contrast, histopathology-based studies, which employ a growing number of immunohistochemistry (IHC) and related methods, now demonstrate key immune biomarkers that in many cases address certain spatial or morphological aspects of the TME. These methods are supported by a variety of descriptive, semiquantitative, and quantitative scoring methods. The ability of histopathology to provide accurate quantitative data in a way that also addresses the spatial aspects of the TME remains limited, though image analysis is increasingly addressing this limitation [9–13]. In addition, the inconsistency with which IHC biomarker analyses are conducted by multiple laboratories hinders the ability to compare histopathological changes across cancer types [14]. Overcoming these challenges requires novel methods and better harmonization among laboratories.

CD8 is a transmembrane glycoprotein expressed mainly on the surface of cytotoxic T lymphocytes. Studies of preclinical models and human cancers have shown that CD8-expressing (CD8+) lymphocytes limit neoplastic cell growth, suppress tumor infiltration, and mediate the outright elimination of tumors [15–17]. Accordingly, CD8 parameters have been assessed in many studies of cancer therapy, including chemotherapy, radiation, and immunotherapy [18–20]. CD8 IHC itself has been used to demonstrate important facets of the immune response to cancer [21–27]. For example, enumeration of CD8+ tumor-infiltrating lymphocytes (TILs) has been shown to be a reliable prognostic marker for a number of cancers, including colorectal cancer and non–small-cell lung carcinoma (NSCLC) [3, 21, 23, 24, 28]. CD8 combined with CD3, CD45RO, FoxP3, granzyme B, or other IHC markers may provide information of even greater prognostic significance [7, 21]. Measuring CD8+ TILS may also help to identify patients who are responsive to cancer immunotherapies. In addition, these and other studies show the importance of immune cell distribution as a measure of the immune response to cancer.

Enumerating CD8+ TILs together with TILs expressing either CD3 or CD45RO serves as the basis of the so-called “immunoscore.” This is a highly reliable prognostic marker in colorectal cancer and is currently being investigated for use in other tumor types [24, 28, 29]. The immunoscore is especially notable because it requires the enumeration of TILs in specific locations in the TME, namely, the tumor center (TC) and invasive margin (IM). A similar approach combining CD8+ TILs with CD163+ macrophages has shown prognostic value in breast cancer patients recently [30]. Other studies have similarly ascribed significance to CD8+ TILs in additional tumor compartments, such as those infiltrating the tumor epithelium or the stroma [26, 31]. Studies have also recently begun to ascribe prognostic significance based on the spatial relationships between immune cells expressing individual markers (eg, CD8 and FoxP3) in different tumor compartments [32]. Thus, CD8 represents a key example of the ways in which histopathology is beginning to address the spatial complexities of the TME as part of our overall understanding of cancer immunity and toward the development of useful biomarkers relevant to the use of immunotherapies.

CD8 IHC further illustrates the limited capacity of histopathology to compare multiple cancers due to the great variability in the methods used by different laboratories. This is exemplified by the use of multiple IHC assays and especially by the use of different means of quantifying CD8 or CD8+ TILs. Some investigators have measured CD8+ TILs using semiquantitative methods [33, 34] or pixel-based measurements with digital image analysis (IA) [35]. Others have digitally or manually counted CD8+ TILs, though typically only in a fraction of tumor sections [16, 36–38]. The original immunoscore itself was used to quantify CD8+ TILs in the TC and IM of colorectal cancer, but only in a few selected regions of each patient sample [29]. Although such methods help us to understand the TME, they do not support comparisons of CD8 results across a range of studies. Studies that instead use IA to quantifiably and reproducibly measure TIL numbers across entire tumor sections are rare [39] and to our knowledge have not been validated for multiple tumor types. In this context, we report the development and validation of digital IA scoring methods that have been customized to profile multiple CD8 measures in whole-tumor sections of diffuse large B-cell lymphoma (DLBCL), gastroesophageal carcinoma (GEC), non-squamous (LNSQ) and squamous (LSCC) types of NSCLC, pancreatic carcinoma (PANC), primary prostate carcinoma (PROS), renal cell carcinoma (RCC), squamous cell carcinoma of the head and neck (HNSCC), and urothelial bladder carcinoma (UBC). We further profiled CD8 in select clinical specimens of NSCLC. Our findings showed a number of differences in CD8 parameters among individual patient samples and between different types of cancer. Such an overall approach can be applied in a common manner to increase the comparability of tumor profiling for CD8 or other immune markers performed by multiple laboratories. The use of validated and harmonized IA methods such as these not only should contribute to our understanding of the immune response to cancer but can also advance the development of tumor biomarkers needed to effectively pair subsets of cancer patients with appropriate immunotherapies.

## Methods

### Study design

We developed sample acquisition, histology, IHC, digital scanning, IA, and related processes to quantitatively assess lymphocytes expressing CD8 in the TME of various cancers that included degrees of spatial and morphological evaluation. Automated IA algorithms were developed to measure multiple CD8 phenotypes in formalin-fixed, paraffin-embedded (FFPE) samples of multiple cancer types, each of which was validated against histological evaluation by pathologists. These methods were applied first to excisional, nonclinical specimens of nine major cancers. Next, we validated this IA process in clinical biopsies of NSCLC patients treated with durvalumab in a phase 1/2 clinical trial (NCT01693562) and compared CD8+ TIL densities in a set of 25 paired biopsy specimens collected at screening and during treatment. We then developed and validated a scoring method to enumerate CD8+ TILS in PD-L1–positive and –negative tumor regions in a separate set of dual-stained clinical specimens. Finally, we applied a novel IA algorithm to detect and quantify elongate TILs, a morphological variant of CD8+ lymphocytes, in the nonclinical samples.

### Sample sets

Up to 50 FFPE blocks of nonclinical, resected tumors were acquired for each cancer type from commercial sources (Asterand Bioscience, Detroit, MI; Analytical Biological Sciences, Wilmington, DE; Avaden Biosciences, Seattle, WA; ILSBio, Chestertown, MD; ProteoGenex, Culver City, CA; Cureline, San Francisco, CA; Tissue Solutions, Glasgow, Scotland; BioServe, Beltsville, MD; Boca Biolistics, Pompano Beach, FL; Conversant Healthcare Systems, Huntsville, AL). Samples were obtained according to sample collection protocols and informed-consent agreements approved by each company’s institutional review board. The numbers of samples that passed all quality control criteria required for data production are shown in Additional file 1: Table S1. Individual samples were selected according to applicable diagnostic criteria and based on histological quality assessment. In each tumor set, we attempted to avoid over-weighting for certain important patient factors (eg, TNM stage, Additional file 1: Table S2). However, given practical limitations, we did not attempt to balance all patient parameters that are potentially relevant to CD8.

Clinical biopsies were obtained as part of a phase 1/2 study to evaluate the safety, tolerability, and pharmacokinetics of durvalumab in subjects with advanced solid tumors (NCT01693562). In part 1, matched pairs of tumor specimens obtained at screening and during therapy (day 43 ± 7) from 25 enrolled NSCLC patients treated with durvalumab were analyzed for CD8 expression. Inclusion of particular patients was based only on paired-specimen availability. Only the fresh biopsy specimen was included for analysis to ensure that it was more temporally comparable to the fresh posttreatment specimen, as well as to more closely match the quality of specimens obtained for the on-drug biopsies (eg, core needle biopsy, more consistent fixation and histological processing). All but one of the 25 subjects had a suitable fresh screening biopsy. In the remaining case, the fresh screening biopsy was not suitable, so the archived sample from this patient (obtained 2 months before the fresh screening specimen) was substituted. In part 2, baseline tumor specimens from 24 patients who did not meet screening criteria in the NCT01693562 study were used to compare IA measures in CD8 single IHC versus CD8/PD-L1 dual IHC. These specimens were preselected histologically to represent a range of CD8 and PD-L1 expression.

### Immunohistochemistry

All tissue blocks were sectioned at 4 μm and mounted on positively charged glass slides. For most nonclinical samples, CD8 IHC was performed using a rabbit monoclonal antibody (clone SP239, catalog no. M5394; Spring Bioscience, Pleasanton, CA), and an automated protocol was performed on a Ventana Discovery Ultra instrument (Roche Diagnostics, Ventana Medical Systems, Tucson, AZ) using 3,3′diaminobenzidine as chromogen applied to sections of LNSQ, RCC, GEC, PROS, and DLBCL samples. Sections of human tumor known to contain abundant CD8+ lymphocytes were used as positive control. A monoclonal rabbit immunoglobulin G isotype antibody (catalog no. 760–1029; Roche Diagnostics) was applied to replicate samples as a negative reagent control. After immunostaining, nuclear counterstaining was performed and then the slides were rinsed and dehydrated and coverslips were applied with a permanent mounting medium.

For the LSCC, UBC, HNSCC, and PANC samples, rabbit clone SP57 (Spring Bioscience) was used on the Discovery instrument under protocol conditions that matched the performance of the SP239 assay. To ensure the comparability of this assay with the SP239 assay, CD8+ lymphocytes were enumerated in common fields of view of serial sections of multiple human tonsils and multiple tumor specimens immunostained with both protocols. The numbers of immunolabeled TILs in the two assays were very similar (Pearson correlation coefficient [PCC] = 0.89). The SP57 protocol was also used to stain the matched sets of NSCLC biopsies from clinical trial NCT01693562. Details of the CD8/PD-L1 dual IHC stain are presented in Additional file 2: CD8/PD-L1 dual IHC.

### Digital scanning and tumor annotation

Immunostained slides were digitally scanned with an Aperio AT turbo scanner (Leica BioSystems, Wetzlar, Germany) at 20X magnification. Digital images were viewed with Aperio ImageScope software version 12.1.0 (Leica BioSystems) or VeriTrova software (Definiens, Munich, Germany).

Images were manually annotated by a pathologist using ImageScope or VeriTrova to denote tumor regions. For nonclinical resection specimens, the IM and TC regions of each image were separately annotated on the hematoxylin-eosin–stained section (KES, PM). The annotations were automatically transferred to the CD8 section using software co-registration and were quality controlled for consistency and completeness. Details of this co-registration process are presented in Additional file 2: Co-registration.

**Fig. 1:**
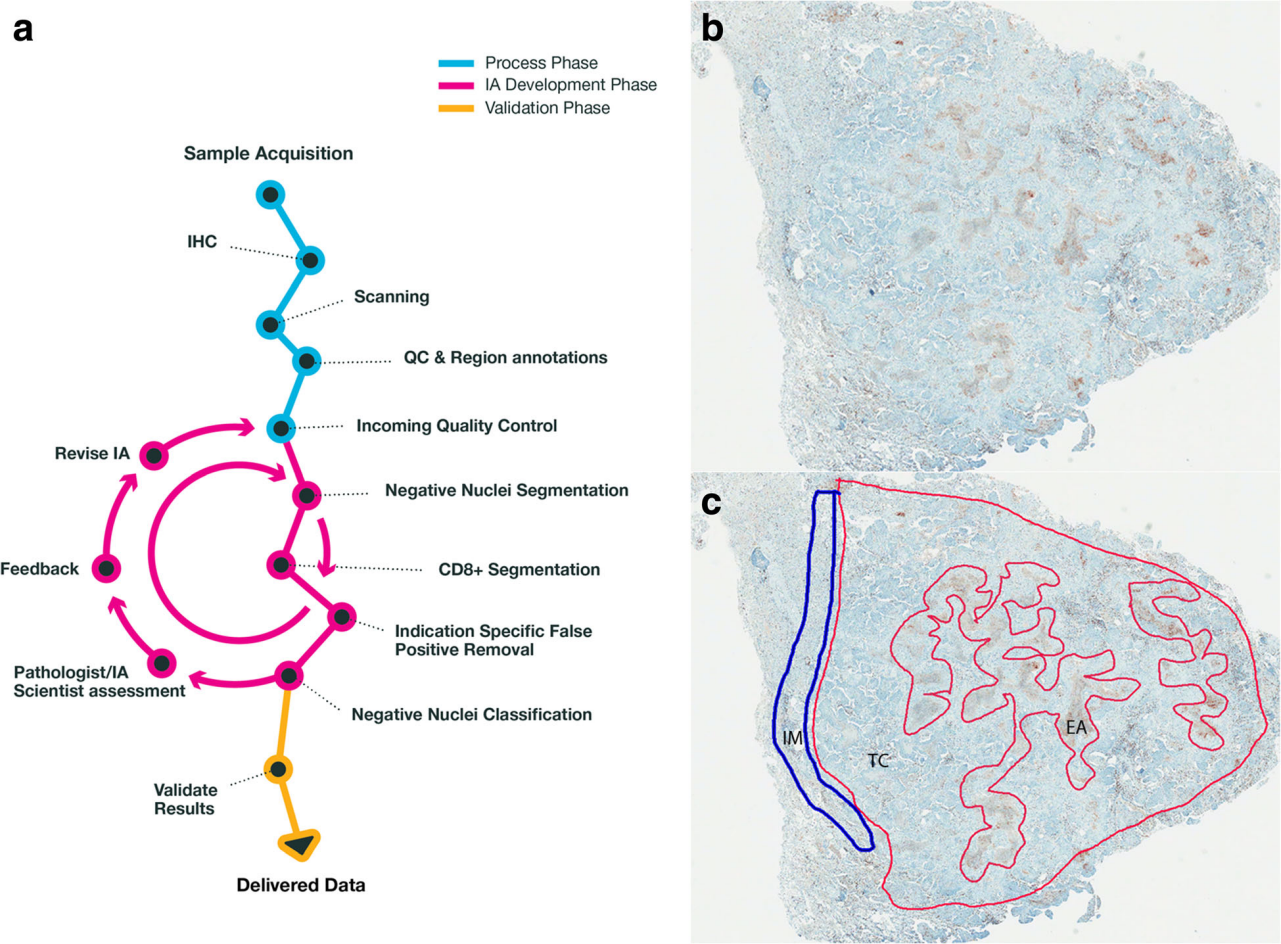
Image analysis (IA) scheme and manual annotations of tumor regions. Key processes in the overall IA scheme leading to data production are depicted (**a**). Tumor regions of images of nonclinical samples were manually annotated by a pathologist to partition invasive margin (IM) from tumor center (TC). Shown is an NSCLC image (**b**) to which annotations (**c**) were applied, along with excluded areas (EA) used to omit necrotic areas or other interfering histological features

The IM annotation represented a band of tissue extending approximately 250 μm beyond and 250 μm inside any tumor margin that was evident microscopically. Some samples did not have a recognizable IM (Additional file 1: Table S1). An IM annotation was not included for DLBCL to avoid uncertainties between tumor-infiltrating cells and resident lymphoid cells along this region. All TC annotations were performed to include as much of the tumor inside the annotated IM as possible. An example of an annotated tumor section is shown in Fig. 1. Because many of the clinical tumor samples were not acquired by resection, and therefore a determination of IM was uncertain for many cases, only a single annotation of “tumor” was performed directly on the CD8 section to differentiate it from adjacent resident tissue or thick stromal bands. This annotated area extended approximately 250 μm beyond any tumor-stroma margin that was evident microscopically whenever sufficient adjacent tissue was present. Substantial areas of necrosis, hemorrhage, or staining and histological artifacts were omitted from analysis either by non-inclusion in the manual annotations or by application of additional exclusion annotations. All digital image files and image annotations were uploaded to Definiens via Aspera Faspex (Emeryville, CA) high-throughput data transfer. A number of quality assessment measures to account for the results of key processes in our overall system were also performed, as described in Additional file 2: Quality control.

### Digital IA scoring methods

The digital images were imported into Developer XD software (Definiens) and analyzed using the program’s cognition network technology [40]. In other studies, different IA methods have been applied to segment immunolabeled cells and hematoxylin-stained nuclei [41–46]. Here, we used rule-based methods combined with machine learning to segment cells and nuclei of acquired images, using Developer XD [47]. This approach was deemed most suitable to account for a number of variables that can influence the quality of CD8-related digital readouts, such as histological quality, tissue necrosis, and immunostaining variability across numerous tumor specimens. To accurately detect CD8+ cells, we translated knowledge from pathologists into a rule-based IA algorithm that is implemented in Developer XD using cognition network technology. First, clearly immunolabeled nonclustered cells were detected, followed by identification of more challenging cell shapes. Then, regions of densely clustered CD8+ lymphocytes were accurately resolved, taking into account more complex local information of the cells judged to be positive. In addition, simple morphological descriptors of valid CD8+ cells, such as size, ellipticity, and compactness, as well as complex features such as completeness of membrane labeling, were optimized. Customized removal of cells that were deemed false positive incorporated adaptive stain-intensity thresholds combined with morphological criteria. For instance, weakly stained or partial cells that were unassociated with a detected nucleus were generally discarded.

Here, we focused on CD8+ cells that were segmented in pathologist-annotated IM and TC regions. To compare the readouts across indications and samples, the data were normalized and reported as density of CD8+ cells per square millimeter of annotated area. We also analyzed the morphological appearance of CD8+ cells and identified elongate TILs. To qualify as elongate, cells must have been isolated from other CD8+ cells and had a width of less than 10 μm and a length-to-width ratio of greater than 2.3. The length and width of cells were defined as the length and width of the oriented bounding box enclosing the segmented cell. The oriented bounding box was obtained as the minimum enclosing box in the orientation of the main axes of the cell. The axis orientations were computed by eigenvalue decomposition of the covariance matrix of the segmented object. Additional details of this process are provided in Additional file 2: Details of digital IA scoring solutions.

### Validation

To validate IA results, we first performed a plausibility check of the IA result data, which tests whether CD8+ TIL counts are non-negative whole numbers, whether areas and densities are non-negative floating-point numbers, and whether missing or duplicate IA result data exist. Next, high-level visual assessment was performed by an IA scientist at 2.5X resolution for each slide to identify major errors in the IA, such as incorrect exclusion of histological artifacts. Next, a tool for automated classification assessment was used to assess the quality of CD8+ TIL classification and counts. For each indication, at least 13 high-magnification fields with a diameter of either 250 or 500 μm were selected by a pathologist and by an IA scientist using VeriTrova. These fields were distributed over at least nine different slides. Additional details of this process are provided in Additional file 2: Details of the automated classification assessment. More than 25,000 CD8+ TILs were annotated within selected fields by one, two, or three independent pathologists. Based on that, Lin concordance correlation coefficient (CCC), PCC, and Spearman rank correlation coefficient (SCC) were calculated. Finally, a pathologist visually inspected select tumor regions that yielded relatively discordant results, using either VeriTrova or Developer XD.

### Statistical analysis

CCC, PCC, and SCC were used to assess the correlation between pathologist annotation and IA classification. To this end, for each sample set, the CD8+ TIL counts per high-magnification field of view that were identified by the IA and by the pathologists were compared against each other. For cell-by-cell assessment, the F_1_ score was used [48]. Here, the sum of true positive, false positive, and false negative across all high-power fields of a sample set was used for calculation. One-sided 95% confidence intervals were calculated using a nonparametric bootstrapping approach with 10,000 iterations [49]. Developer XD software (Definiens); in-house software; R, an open-source software environment for statistical computing and graphics [50]; and the packages epiR [51], gdata [52], scales [53], gtools, and ggplot2 [54] were used to compare annotations against CD8+ TILs classified by IA.

Statistical analysis for the comparison of cell densities across indications and between TC and IM was carried out and plotted in R. The nonparametric Wilcoxon signed-rank test with a testing level α of 0.05 was performed for the paired comparison between the cell densities in TC and IM, and the test statistics were indicated as Wilcoxon T, using *T* < 0.05 to signify statistical significance. Detailed information regarding the data used for statistical analyses are shown in Additional file 1: Table S3.

## Results

### IA scheme and scoring validation

We used a number of means to process and optimize the quality of immunolabeled specimens and to assess the accuracy of our IA results in both clinical and nonclinical tumor specimens. The key elements of our IA scheme are summarized in Fig. 1a. Only images that passed quality control checks were included in subsequent IA (Additional file 1: Table S1). For suitable images of nonclinical samples, a pathologist (KES, PM) manually annotated the IM and TC regions of each of these tumors (Fig. 1). This permitted the partitioning of CD8 results according to IM, TC, tumor area (IM and TC combined), or full section to include any adjacent resident (non-tumor) tissue. For clinical trial specimens, tumor regions were annotated with no distinction between IM and TC. This was done because many clinical specimens were needle biopsy cores and it was often not possible to histologically distinguish an apparent IM.

**Fig. 2:**
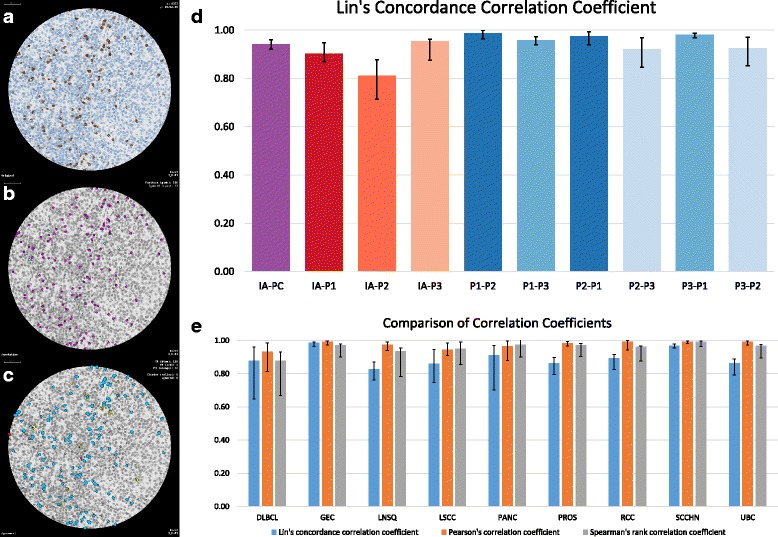
Validation of image analysis (IA) classification and enumeration of CD8+ TILs. Unclassified images (**a**) were examined by pathologists at high magnification, and immunolabeled TILs were annotated in purple (**b**). The IA software then characterized each cell (**c**) as true positive (blue), false positive (red), or false negative (orange). For clinical trial NCT01693562, concordance between IA and each of three pathologists (**d**) was determined. For nonclinical samples, concordance between IA and one pathologist was determined and compared, using three statistical measures for all nine tumor types (**e**)

Images of full tissue sections were processed with IA methods that automatically detected the tissue, loaded the pathologist’s annotations, and segmented CD8+ cells. Classified image results were reviewed visually by a pathologist and compared with the unclassified IHC images (Additional file 3: Figure S1) to initially assess the accuracy of the CD8 detection. The numbers of CD8+ lymphocytes determined by IA were then compared with those of pathologists for all sample sets in a blinded fashion. We found that the majority of CD8+ lymphocytes that were evident microscopically were detected by IA and annotated by pathologists (Fig. 2a–c). The overall numbers of immunolabeled TILs counted by IA and pathologists showed good concordance by multiple statistical measures (Fig. 2d, e). In particular, we emphasized the CCC [55] over the PCC [56] or the SCC [57]. Although PCC and SCC are blind with respect to systematic over- or under-counting, the CCC is not. Therefore, CCC is better suited to gain confidence in the comparability of results. To characterize the strength of agreement, we applied the criteria proposed by McBride [58]. Accordingly, a lower one-sided 95% confidence interval of the CCC (CCC_lower) of ≥0.9 was characterized as almost perfect, ≥0.8 as substantial, ≥0.65 as moderate, and < 0.65 as poor agreement.

Our validation of IA applied to clinical and nonclinical sample sets varied slightly (Fig. 2d, e). For the matched biopsy sets from NSCLC patients in the phase 1/2 clinical trial, we originally compared cell annotations acquired by a single pathologist with the IA results shown here. Based on the CCC, the agreement between IA and pathologist was almost perfect (CCC = 0.97, CCC_lower = 0.94). To also understand the impact of scoring by different pathologists, we compared cell annotations that had been acquired independently by three pathologists. The agreement between pathologists was substantial to almost perfect (CCC = 0.92–0.99, CCC_lower = 0.85–0.97). For this dataset, we also validated an updated version of the IA method. The applicable IA revisions were minor and included a software update, speed improvements, and exclusion of negative cell detection because that part of the scoring solution had no effect on determination of CD8+ TIL density. Agreement between both IA and all three pathologists (KES, PM, JZ) individually (CCC = 0.0.81–0.96, CCC_lower = 0.68–0.91), as well as agreement between IA and consolidated pathologist scores (CCC = 0.94, CCC_lower = 0.92) were deemed between moderate and almost perfect. For the nonclinical sets, IA was compared with pathologist scores from a single pathologist for each tumor type. There was also good concordance between IA and pathologist scores for the multiple tumor sets, although somewhat greater differences were evident for CCC than for PCC or SCC. For all indications, CCC showed at least moderate agreement with the pathologist annotations (CCC = 0.88–0.99, CCC_lower = 0.65–0.96). Finally, our validation process, which was carried out in the course of developing the scoring method, included a visual inspection of tumor regions that yielded relatively discordant results. We found that these regions, such as in the IM of multiple cases of GEC, contained tightly packed lymphocytes that were not microscopically identifiable as single cells. In essence, neither the IA method nor the pathologist could accurately assess the number of CD8+ lymphocytes in such foci. We also observed small regions of immunolabeled partial cells with nuclear debris, ie, apparent lymphocytolysis, that neither the manual annotations nor the IA filtered out. These too yielded inconsistent agreement between the pathologist and IA. Because these findings typically occurred in regions of high CD8+ TIL density and were relatively infrequent, such problems did not appear to affect the accuracy of CD8 readouts.

### Validation of IA scoring of a CD8/PD-L1 dual assay and detection of elongate CD8+ lymphocytes

**Fig. 3:**
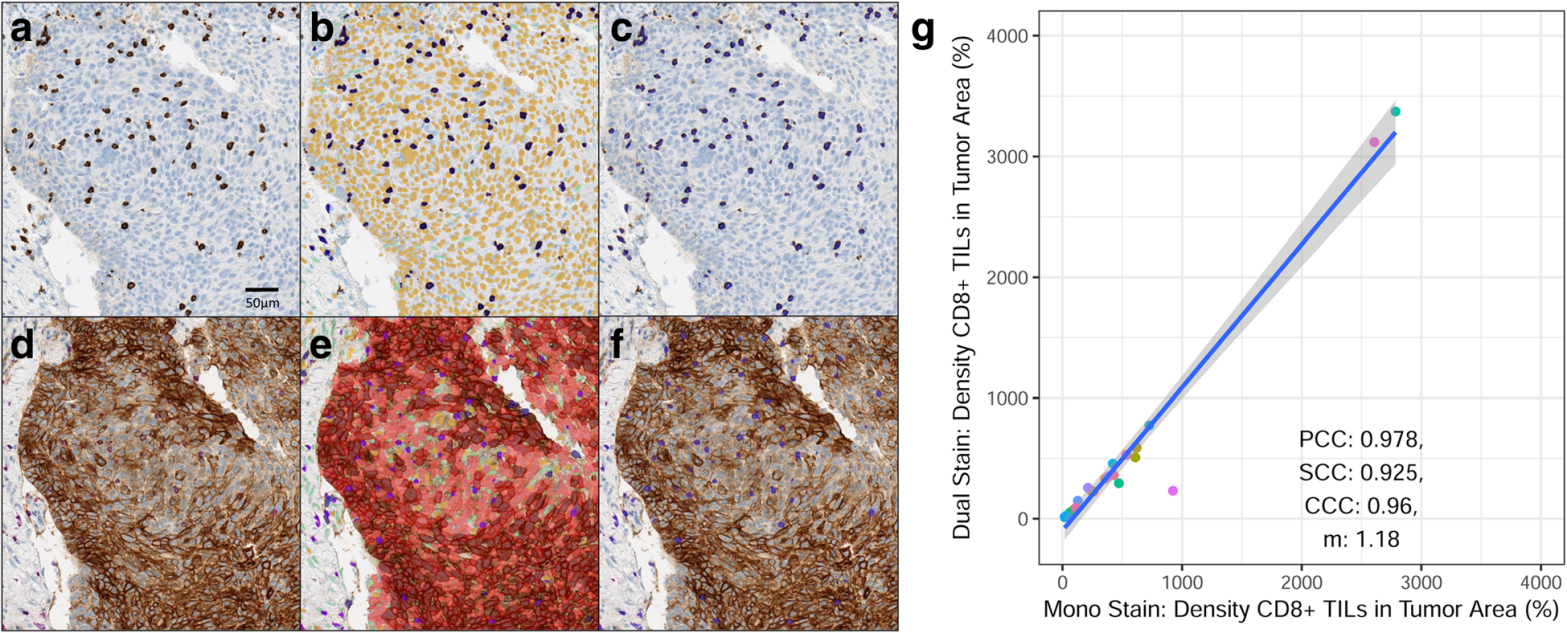
Image analysis of CD8+ TILs in PD-L1–positive and –negative tumor. Serial sections of tumor specimens of 24 non-enrolled NSCLC patients of clinical trial NCT01693562 were immunostained for CD8 alone (**a**, **b**, **c**) and with a CD8/PD-L1 dual immunostain (**d**, **e**, **f**). CD8+ TILs were immunolabeled brown in the mono stain (**a**) and purple in the dual stain (**d**), with PD-L1 labeled brown. IA detected CD8+ TILs as blue in the mono stain (**b**) and lavender in the dual stain (**e**). IA further classified tumor cells (**c**, yellow areas) in the mono stain or PD-L1+ cells (**f**, red areas) in the dual stain; darker shades of red represent more intense PD-L1 expression. IA determined the numbers of CD8+ TILs in the two stains to be comparable (**g**); Pearson (PCC), Spearman (SCC), and Lin (CCC) concordance values are shown

Recognizing the potential for IA to measure histological features beyond simple enumeration of conventional CD8+ TILs, we tested the strength of additional CD8 parameters of possible relevance to the immune response to cancer. First, we examined CD8 IHC in the context of PD-L1, which is foremost among the immunosuppressive molecules currently under investigation. The relationship between CD8+ TILs and tumor expression of PD-L1 has recently become an area of interest [59, 60]. To begin to study the spatial relationship between CD8+ TILs and PD-L1–positive and –negative tumors, we applied a dual immunostain to NSCLC samples and then evaluated the ability of IA to accurately enumerate CD8+ TILs. In a group of 24 NSCLC tumor samples of screened patients who did not meet the criteria for enrollment in clinical trial NCT01693562, the IA results showed that the overall numbers of CD8+ TILs detected in the double stain were highly comparable to those detected in the CD8 single stain (Fig. 3). We also found that the IA scoring method showed good concordance values with scoring by two pathologists (Additional file 2: Measurement of CD8+ TILs in PD-L1–positive tumor and Additional file 1: Table S4). We concluded that our IA could accurately measure CD8+ TILs using this double IHC method and that the CD8 part of the dual stain was comparable in sensitivity to that of the single IHC. These findings support the use of IA to explore spatial relationships involving CD8+ lymphocytes and PD-L1 in the TME.

Microscopic analysis of numerous CD8-immunostained tumors revealed that some CD8+ TILs were elongate and thus morphologically distinct from the generally round shape of most TILs. On the basis of in vitro studies of cancer cell killing showing that motile cytotoxic T lymphocytes are elongate, whereas sessile lymphocytes are rounded [61], we explored the ability of IA to detect elongate CD8+ TILs in tissue sections as a potential marker of TIL motility. To this end, an IA rule set was developed to automatically discriminate and enumerate elongate CD8+ TILs from non-elongate TILs. Visually, the IA results showed proper classification of elongate cells as separate from CD8+ TILs of conventional shape in the majority of instances (Additional file 3: Figure S2). To rule out the possibility that these cells could be reactive fibroblasts or macrophages, we also showed that elongate CD8+ TILs expressed CD3 by double immunofluorescence staining in nearly all instances (Additional file 3: Figure S3). We then applied this algorithm to the nine tumor sets. The resulting data showed that elongate CD8+ TILs represented a small fraction of overall CD8+ TILs (Additional file 3: Figure S4), consistent with histological evaluation, as well as some differences in percentages of elongate TILs among tumor types. Importantly, it remains to be proven that this novel morphological signature specifically measures motile CD8+ lymphocytes in tumor tissues. The IA algorithm we developed should, however, provide a useful analytical tool to investigate this possibility.

### Comparison of CD8+ TIL densities across cancer indications and between tumor regions

**Fig. 4:**
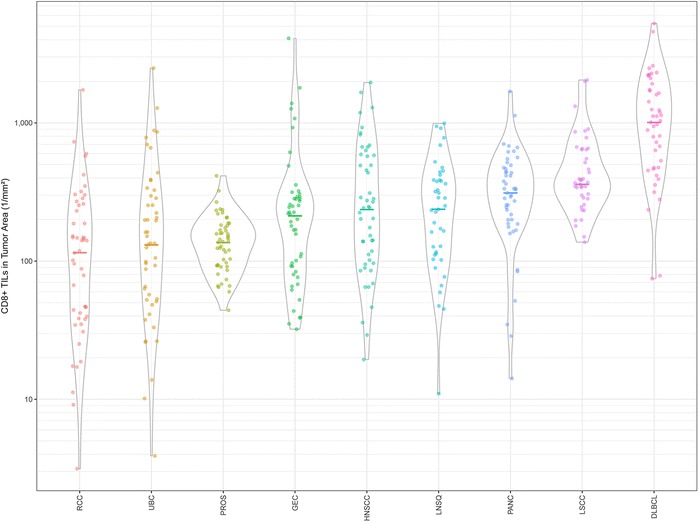
CD8 tumor landscape. The density of CD8+ TILs in the tumor area (tumor center and invasive margin combined) of individual nonclinical specimens are shown as dots and grouped as violin plots for each cancer indication. The median density of CD8+ TILs is also shown (bars) for each indication

Numerous cancer studies have confirmed the importance of CD8 as a principal intratumoral marker of immune activity [4, 15]. In that light, and supported by our IA validation results, a major goal of this study was to compare CD8+ TILs between individual samples and across tumor types. These data for tumor area (IM and TC combined) are shown in Fig. 4. Notably, the median densities of CD8+ TILs varied among the different tumor types. DLBCL had the highest median number of CD8+ TILs, several-fold higher than those of RCC, UBC, and PROS. It is not surprising to see high numbers of CD8+ TILs in DLBCL, given that B-cell lymphomas commonly contain non-neoplastic T lymphocytes that outnumber the neoplastic lymphocytes themselves [62]. Our data also show a range of CD8+ TIL densities among individual patient tumors for DLBCL and most of the remaining cancer types. This is not unexpected, as several studies have shown variability in TIL numbers among individual patients affected by particular types of cancer [3, 21, 23, 24]. Thus, as in the case of RCC, although this tumor type had the overall lowest median density and a number of individual samples that were extremely low, other RCC tumors instead had CD8+ TILs well above the median for all other tumor types except DLBCL. By comparison, PROS had a relatively low median density and individual tumors demonstrated a narrow range of CD8+ TILs. LSCC and LNSQ had relatively high median CD8+ TIL densities. LNSQ, however, demonstrated a larger proportion of low individual values, similar to PANC, HNSCC, and GEC. Although contrasts among tumor types such as these seem interesting, further study is needed both to demonstrate consistency in larger sample sets and to possibly begin to explain the reasons for these differences.

**Fig. 5:**
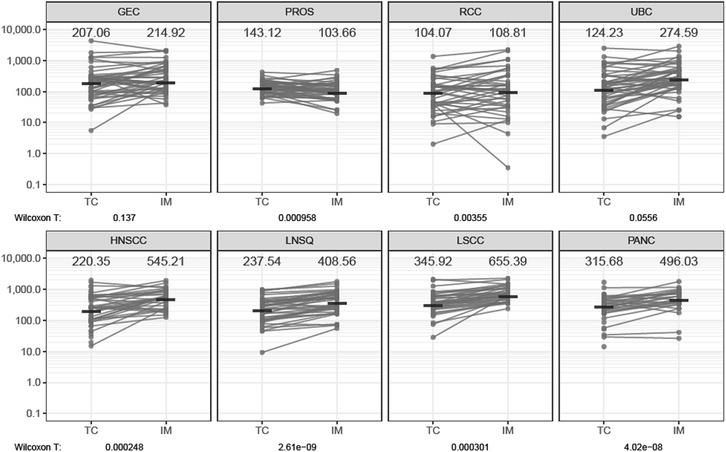
Paired density plot of CD8+ TILs in tumor center (TC) and invasive margin (IM) across cancer indications (1/mm^2^). CD8+ TILs in the TC and IM were compared. For each nonclinical specimen, CD8+ TIL densities were determined separately in annotated tumor regions as shown in Fig. 1c. Individual TC scores are plotted as dots and connected to the applicable IM score by a line. Median values for each are shown. For each tumor type, the Wilcoxon T values denote the degree of statistical difference between TC and IM CD8+ TIL densities. Some specimens without an identifiable IM are represented by isolated dots for TC (eg, pancreatic carcinoma). DLBCL is not shown because CD8+ TILs were not enumerated in the IM, as explained in the text

Our use of manual annotations permitted additional comparisons based on tumor region (Fig. 5). Notably, CD8+ TILs in the IM were significantly more numerous than in the TC for LSCC, LNSQ, HNSCC, RCC, and PANC. The reverse (TC > IM) was significantly different only for PROS. In individual cases representing all indications, the differences in CD8+ TILs between IM and TC varied from modest to several-fold. It is possible that differences in the efficiency of TIL trafficking within particular regions of certain tumors could help explain such patterns.

### CD8+ TIL densities in matched biopsy sets of patients treated with durvalumab

**Fig. 6:**
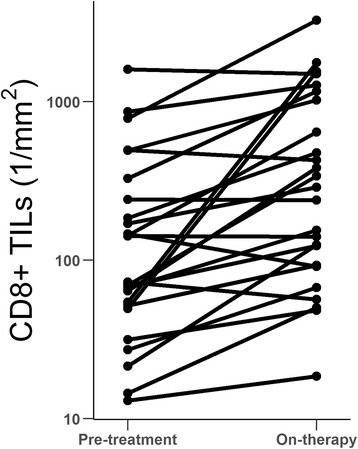
CD8+ TIL densities as a measure of durvalumab pharmacodynamic activity in clinical trial NCT01693562. Enumeration of CD8+ TILs by image analysis was performed on matched sets of pretreatment and on-therapy (±6 weeks) specimens of 25 NSCLC patients. Tumor was manually annotated by a pathologist. The density of CD8+ TILs in paired sets was compared using a two-sided paired *t* test. Of 25 patients, 20 had increased CD8+ TILs during therapy, with an average increase of 365 cells/mm^2^ (*P* = 0.009, 95% confidence interval = 101.3–628.5)

We assessed densities of CD8+ TILs as a potential pharmacodynamic marker in a set of 25 paired biopsy specimens obtained at baseline and during therapy from NSCLC patients in the phase 1/2 clinical trial investigating the PD-L1 antibody therapy durvalumab (NCT01693562) [63]. Tumor specimens acquired during treatment were collected approximately 6 weeks after the initial dose of durvalumab. Of the 25 included patients, 20 had more CD8+ TILs during therapy than at baseline (Fig. 6). An average increase of 365 cells/mm^2^ over baseline was observed in the on-therapy specimens (*P* = 0.009). These findings were interpreted as evidence of immune activation consistent with the mechanism of action of durvalumab. It is noted that this sample set was not sufficiently powered to make meaningful comparisons in CD8+ TIL densities between clinical responders and nonresponders.

## Discussion

Studies using CD8 IHC have contributed to our understanding of cancer immunity in important ways. Key among these is the clinical consequence of CD8+ TILs in human cancer patients, as discussed in a recent comprehensive review of major types of cancer in which CD8 was considered alongside other T-lymphocyte measures [5]. Although very informative, reports such as this also serve to illustrate the limitations of CD8-related studies. For example, quantitative CD8 IHC approaches often lack the ability to accurately analyze large sets of whole-tumor sections, use different means of scoring CD8 TILs, and frequently have uncertain validation of the IA methodology when used. This overall lack of harmonization, in particular, limits the ability to compare data across sample sets. In this context, we report the development and validation of quantitative, whole-image IA to measure multiple CD8 parameters in the TME of clinical and nonclinical specimens. Such methods in general should increase the overall reliability and comparability of tissue-based, quantitative IHC data. CD8 tumor measures specifically, whether alone or combined with other immune markers, should continue to provide important translational value to the field of immuno-oncology.

The ability to compare CD8+ TILs across multiple types of cancer by IHC based on a harmonized approach has not previously been reported. Thus, the data from our analysis of nonclinical samples provides a potentially helpful comparative view of the immune response to cancer. For instance, the different levels of CD8+ TIL densities that are evident among various cancers may serve as an indicator of the relative immune responsiveness of each tumor type, based on concepts of CD8 previously proposed [5, 15–17, 19, 20]. In that regard, we found the highest numbers of CD8+ TILs in DLBCL specimens, providing additional support for the prevailing notion of DLBCL as an immunogenic cancer. Based on the additional belief that immunogenic types of cancer are potentially amenable to checkpoint immunotherapy, it is worth noting that a recent study showed meaningful response to the PD1 antibody nivolumab in patients with DLBCL [64]. By comparison, we found RCC and UBC had much lower CD8 group values, suggesting that they may be relatively less immune responsive. However, such an interpretation could be misleading, because a number of individual RCC and UBC samples had relatively high CD8+ TIL densities. These findings are consistent with the meaningful response to immunotherapies experienced by some patients with these cancers. In contrast, PROS samples demonstrated a low median value within a narrow range of individual CD8 TIL densities. Combined, these findings more strongly support the notion of PROS as an immunosuppressed tumor type, and one for which multiple immunotherapy approaches have failed [65]. Another notable finding in our data is the significantly higher CD8+ TIL densities in the IM versus the TC for multiple cancers. Only PROS samples demonstrated a significantly lower density of CD8+ TILs in the IM than in the TC. We are not aware of any basis for such a distinction in primary PROS, although one possibility is that TIL trafficking patterns might somehow be different in this versus the other tumors. Additional interpretations of the CD8 data presented here are possible.

There are reasons to interpret our data with some caution. For example, the data representing the CD8 tumor landscape were based on a maximum 50 samples of each tumor type, which may not reflect the overall population for the cancer type represented by each sample set. The reasons for potential bias in small sample sets could be related to a number of factors that may affect CD8. These might include mutations in molecules such as epithelial growth factor receptor, microsatellite instability, or additional molecular elements that represent the overall complexity of cancer. In particular, a degree of possible bias might pertain to the RCC samples in our study. We compared the patterns evident in our CD8 tumor landscape (Fig. 4) to a similar multitumor comparison based on TCGA data [66]. We found that DLBCL had high TIL densities in both data sets, PROS was low in both, and other cancers were in between. However, RCC represented an exception. The TCGA Kidney Renal Clear Cell Carcinoma database demonstrated a relatively high TIL score, yet in our IHC set, RCC had the lowest median value of CD8+ TILs. In view of the larger number of samples in the TCGA comparison, it is possible that our RCC set included a comparatively greater proportion of CD8-low specimens. Otherwise, the TCGA data tended to corroborate the relative TIL group values that we found. Analysis of larger tumor sets in general should provide a more accurate view of the CD8 landscape. Analysis of particular molecular subsets of cancers, or additional sets accompanied by important patient information (eg, survival) would also be valuable.

It is also important to interpret our data with the understanding that some tumors harbor CD8+ TILs weighted toward a dysfunctional, rather than functional, immune-responsive phenotype [15, 28]. Measuring TIL densities based on CD8 IHC alone may therefore be a misleading indicator of immune activity in individual cases. In that regard, the use of multiplex immunostaining is increasingly being used to better define the phenotype of individual CD8+ TILs, to study the topographical relationships between CD8 and other immune cells, or to determine the localization of CD8+ TILs in distinct compartments (eg, desmoplastic stroma) [10–13]. Adding quality IA methods to multiplex immunostaining should provide a powerful combination with the ability to better understand the context of CD8+ TIL infiltrates or the immune response to cancer. We are currently investigating several additional immunoregulatory proteins by IHC or multiplex immunostaining in many of the sample sets reported here, using an IA approach similar to that used for CD8. This should expand our classification of individual tumors and provide helpful immunological context with which to interpret the CD8 data presented here. Appropriate gene expression analysis of these same FFPE tissues could provide additional value.

Our inclusion of tumor samples from patients with NSCLC enrolled in the NCT01693562 trial allowed us to test our IA approach in clinical biopsy specimens. The resulting high concordance between IA and pathologist scoring of CD8 supports the applicability of our methods to accurately measure CD8 in clinical samples as a marker of immune activation. We used this finding to show drug activity in patients treated with durvalumab, consistent with the mechanism of action of this PD-L1 antibody.

Finally, we extended our IA approach to quantify conventional CD8+ TIL parameters to demonstrate the ability of IA to also capture more complex histopathological information. In one way, we developed a scoring algorithm that discriminates and quantifies elongate CD8+ TILs as a morphological parameter that could serve as a measure of TIL motility, as suggested by in vitro studies [61]. This is relevant to immuno-oncology based studies that recently linked increased motility of cytotoxic T lymphocytes to the efficiency of target cell killing [67] and separately hypothesized that TIL motility might help explain the activity of antibody therapy to CTLA-4 [68]. We found elongate TILs detected by IA to be consistent with microscopic assessment of TIL shapes. We also found the resulting IA data to be further consistent with our observation that elongate CD8+ TILs represented a fraction of overall CD8+ TILs in all cancers studied. It remains to be demonstrated, however, that elongate TILs in tissues are in fact motile TILs. It also remains to be shown that this kind of morphological signature has any clear functional relevance to the immune response to cancer. We are addressing these and other potentially relevant characteristics of elongate TILs in our ongoing studies. A second forward-looking aspect of IA we tested was to demonstrate the accuracy of CD8+ TIL detection using a dual CD8/PD-L1 IHC assay. We consider this an important practical consideration related to the current idea that scoring subsets of cancers according to TIL infiltrates and PD-L1 expression is a useful stratification scheme for immunotherapies targeting PD1/PD-L1 [28, 61]. It also extends our previous work showing that a signature of CD8+ TIL densities combined with PD-L1+ cell densities measured in separate sections of NSCLC biopsies afforded a greater ability to predict response to durvalumab than either measure alone [69]. Our validation of CD8+ TIL assessment in this way supports our ongoing studies to quantify spatial relationships between CD8+ TILs and PD-L1+ cells in the TME. It also illustrates, for CD8 in the context of PD-L1, the kind of validation of individual IA parameters needed to support the broader use of IA to measure complex spatial and morphological aspects of the TME.

## Conclusions

Validated image analysis accurately measures CD8+ TILs in histological sections of human cancers. The quantitative data thus presented here provide comparative CD8 baseline information not previously reported for several common types of cancer. Automated IA should strengthen the ability of CD8 to serve as a pharmacodynamic or predictive marker of relevant immunotherapies. The use of validated and harmonized IA methods moreover should contribute to our understanding of the immune response to cancer. It also has the potential to speed efforts to develop the tumor biomarkers needed to advance cancer immunotherapy.

## Additional files

Additional file 1: **Table S1** Relevant image information for nonclinical sample sets. **Table S2** Nonclinical tumor samples, key patient data.**Table S3** Overview of data used for statistical analysis. **Table S4** Validation of CD8 IA using CD8 single stain and CD8/PD-L1 dual stain. (DOCX 91 kb)

Additional file 2: CD8/PD-L1 dual IHC, quality control; co-registration; details of the automated classification assessment; details of digital IA scoring solutions; measurement of CD8+ TILs in PD-L1–positive tumor. (DOCX 45 kb)

Additional file 3: **Figure S1** IA classification of CD8+ lymphocytes. **Figure S2** IA classification of elongate CD8+ lymphocytes. **Figure S3** Elongate CD8+ TILS detected by IA are also CD3+. **Figure S4** Tumor landscape of elongate CD8+ TILs. (DOCX 1795 kb)

## Abbreviations

CCC: Lin concordance correlation coefficient
CCC_lower: lower one-sided 95% confidence interval of the Lin concordance correlation coefficient
DLBCL: Diffuse large B-cell lymphoma
F1: F1 score
F1_lower: lower one-sided 95% confidence interval of the F1 score
FFPE: Formalin-fixed, paraffin-embedded
GEC: Gastroesophageal carcinoma
HNSCC: Head and neck squamous cell carcinoma
IA: Image analysis
IHC: Immunohistochemistry
IM: Invasive margin
LNSQ: non-squamous non–small-cell lung carcinoma
LSCC: squamous-cell non–small-cell lung carcinoma
NSCLC: Non-small–cell lung carcinoma
PANC: Pancreatic carcinoma
PCC: Pearson correlation coefficient
PCC_lower: lower one-sided 95% confidence interval of the Pearson correlation coefficient
PROS: Prostatic carcinoma
RCC: Renal cell carcinoma
SCC: Spearman rank correlation coefficient
SCC_lower: lower one-sided 95% confidence interval of the Spearman correlation coefficient
TC: Tumor center
TCGA: The Cancer Genome Atlas
TIL: Tumor-infiltrating lymphocyte
TME: Tumor microenvironment
UBC: Urothelial bladder carcinoma

## References

[1] Leventakos K, Mansfield AS (2016). Advances in the treatment of non-small cell lung cancer: focus on nivolumab, pembrolizumab, and atezolizumab. BioDrugs.

[2] Tang T, Eldabaje R, Yang L (2016). Current status of biological therapies for the treatment of metastatic melanoma. Anticancer Res.

[3] Topalian SL, Taube JM, Anders RA, Pardoll DM (2016). Mechanism-driven biomarkers to guide immune checkpoint blockade in cancer therapy. Nat Rev Cancer.

[4] Chen DS, Mellman I (2017). Elements of cancer immunity and the cancer-immune set point. Nature.

[5] Fridman WH, Pages F, Sautes-Fridman C, Galon J (2012). The immune contexture in human tumours: impact on clinical outcome. Nat Rev Cancer.

[6] Khalil DN, Smith EL, Brentjens RJ, Wolchok JD (2016). The future of cancer treatment: immunomodulation, CARs and combination immunotherapy. Nat Rev Clin Oncol.

[7] Gnjatic S, Bronte V, Brunet LR (2017). Identifying baseline immune-related biomarkers to predict clinical outcome of immunotherapy. J Immunother Cancer..

[8] National Human Genome Research Institute. The Cancer Genome Atlas. 2017; https://cancergenome.nih.gov/abouttcga. Accessed 5 May 2017.

[9] Caie PD, Zhou Y, Turnbull AK, Oniscu A, Harrison DJ (2016). Novel histopathologic feature identified through image analysis augments stage II colorectal cancer clinical reporting. Oncotarget.

[10] Carey CD, Gusenleitner D, Lipschitz M (2017). Topological analysis reveals a PD-L1 associated microenvironmental niche for reed-Sternberg cells in Hodgkin lymphoma. Blood.

[11] Carstens JL, Correa de Sampaio P, Yang D (2017). Spatial computation of intratumoral T cells correlates with survival of patients with pancreatic cancer. Nat Commun.

[12] Feng Z, Bethmann D, Kappler M, et al. Multiparametric immune profiling in HPV- oral squamous cell cancer. JCI Insight. 2017;2(14):e93652. 10.1172/jci.insight.93652. 10.1172/jci.insight.93652 PMC5518563 28724788

[13] Gorris MAJ, Halilovic A, Rabold K (2018). Eight-color multiplex immunohistochemistry for simultaneous detection of multiple immune checkpoint molecules within the tumor microenvironment. J Immunol.

[14] Rimm DL, Han G, Taube JM (2017). A prospective,multi-institutional, pathologist-based assessment of 4 immunohistochemistry assays for PD-L1 expression in non–small-cell lung cancer. JAMA Oncol.

[15] Apetoh L, Smyth MJ, Drake CG (2015). Consensus nomenclature for CD8+ T cell phenotypes in cancer. Oncoimmunology.

[16] Jackute J, Zemaitis M, Pranys D (2015). The prognostic influence of tumor infiltrating Foxp3(+)CD4(+), CD4(+) and CD8(+) T cells in resected non-small cell lung cancer. J Inflamm.

[17] Koebel CM, Vermi W, Swann JB (2007). Adaptive immunity maintains occult cancer in an equilibrium state. Nature.

[18] Hay CM, Sult E, Huang Q (2016). Targeting CD73 in the tumor microenvironment with MEDI9447. Oncoimmunology..

[19] Lee Y, Auh SL, Wang Y (2009). Therapeutic effects of ablative radiation on local tumor require CD8+ T cells: changing strategies for cancer treatment. Blood.

[20] Mattarollo SR, Loi S, Duret H, Ma Y, Zitvogel L, Smyth MJ (2011). Pivotal role of innate and adaptive immunity in anthracycline chemotherapy of established tumors. Cancer Res.

[21] Ascierto PA, Capone M, Urba WJ (2013). The additional facet of immunoscore: immunoprofiling as a possible predictive tool for cancer treatment. J Transl Med.

[22] Boheim K, Denz H, Boheim C, Glassl H, Huber H (1987). An immunohistologic study of the distribution and status of activation of head and neck tumor infiltrating leukocytes. Arch Otorhinolaryngol.

[23] Dieu-Nosjean MC, Giraldo NA, Kaplon H, Germain C, Fridman WH, Sautes-Fridman C (2016). Tertiary lymphoid structures, drivers of the anti-tumor responses in human cancers. Immunol Rev.

[24] Galon J, Pages F, Marincola FM (2012). The immune score as a new possible approach for the classification of cancer. J Transl Med.

[25] Marrogi AJ, Munshi A, Merogi AJ (1997). Study of tumor infiltrating lymphocytes and transforming growth factor-beta as prognostic factors in breast carcinoma. Int J Cancer.

[26] Naito Y, Saito K, Shiiba K (1998). CD8+ T cells infiltrated within cancer cell nests as a prognostic factor in human colorectal cancer. Cancer Res.

[27] Teng MW, Ngiow SF, Ribas A, Smyth MJ (2015). Classifying cancers based on T-cell infiltration and PD-L1. Cancer Res.

[28] Bethmann D, Feng Z, Fox BA (2017). Immunoprofiling as a predictor of patient’s response to cancer therapy-promises and challenges. Curr Opin Immunol.

[29] Pages F, Berger A, Camus M (2005). Effector memory T cells, early metastasis, and survival in colorectal cancer. N Engl J Med.

[30] Fortis SP, Sofopoulos M, Sotiriadou NN (2017). Differential intratumoral distributions of CD8 and CD163 immune cells as prognostic biomarkers in breast cancer. J Immunother Cancer..

[31] Bottai G, Raschioni C, Losurdo A (2016). An immune stratification reveals a subset of PD-1/LAG-3 double-positive triple-negative breast cancers. Breast Cancer Res.

[32] Feichtenbeiner A, Haas M, Buttner M, Grabenbauer GG, Fietkau R, Distel LV (2014). Critical role of spatial interaction between CD8(+) and Foxp3(+) cells in human gastric cancer: the distance matters. Cancer Immunol Immunother.

[33] Muller P, Rothschild SI, Arnold W (2016). Metastatic spread in patients with non-small cell lung cancer is associated with a reduced density of tumor-infiltrating T cells. Cancer Immunol Immunother.

[34] Park JH, Powell AG, Roxburgh CS, Horgan PG, McMillan DC, Edwards J (2016). Mismatch repair status in patients with primary operable colorectal cancer: associations with the local and systemic tumour environment. Br J Cancer.

[35] Baine MK, Turcu G, Zito CR (2015). Characterization of tumor infiltrating lymphocytes in paired primary and metastatic renal cell carcinoma specimens. Oncotarget.

[36] Djenidi F, Adam J, Goubar A (2015). CD8+CD103+ tumor-infiltrating lymphocytes are tumor-specific tissue-resident memory T cells and a prognostic factor for survival in lung cancer patients. J Immunol.

[37] Mella M, Kauppila JH, Karihtala P (2015). Tumor infiltrating CD8+ T lymphocyte count is independent of tumor TLR9 status in treatment naive triple negative breast cancer and renal cell carcinoma. Oncoimmunology.

[38] Zhu J, Wen H, Ju X, Bi R, Zuo W, Wu X (2017). Clinical significance of programmed death ligand 1 and intra-tumoral CD8+ T lymphocytes in ovarian carcinosarcoma. PLoS One.

[39] Halama N, Michel S, Kloor M (2011). Localization and density of immune cells in the invasive margin of human colorectal cancer liver metastases are prognostic for response to chemotherapy. Cancer Res.

[40] Baatz M, Zimmermann J, Blackmore CG (2009). Automated analysis and detailed quantification of biomedical images using Definiens cognition network technology. Comb Chem High Throughput Screen.

[41] Arteta C, Lempitsky V, Noble JA, Zisserman A (2012). Learning to detect cells using non-overlapping extremal regions. Med Image Comput Comput Assist Interv.

[42] Chen T, Chef'dhotel C, Wu G, Zhang D, Zhou L (2014). Deep learning-based automatic immune cell detection for immunohistochemistry images. Machine learning in medical imaging.

[43] Mualla F, Scholl S, Sommerfeldt B, Maier A, Hornegger J (2013). Automatic cell detection in bright-field microscope images using SIFT, random forests, and hierarchical clustering. IEEE Trans Med Imaging.

[44] Niazi MKK, Satoskar AA, Gurcan MN. An automated method for counting cytotoxic T-cells from CD8 stained images of renal biopsies. Proccedings Volume 8676, Medical Imaging 2013: Digital Pathology; 867606. 10.1117/12.2007977. Available at https://www.spiedigitallibrary.org/conference-proceedings-of-spie/8676/867606/An-automated-method-for-counting-cytotoxic-T-cells-from-CD8/10.1117/12.2007977.short?SSO=1.

[45] Parvin B, Yang Q, Han J, Chang H, Rydberg B, Barcellos-Hoff MH (2007). Iterative voting for inference of structural saliency and characterization of subcellular events. IEEE Trans Image Process.

[46] Xin Q, Xing F, Foran DJ, Yang L (2012). Robust segmentation of overlapping cells in histopathology specimens using parallel seed detection and repulsive level set. IEEE Trans Biomed Eng.

[47] Brieu N, Pauly O, Zimmermann J, Binnig G, Schmidt G (2016). Slide-specific models for segmentation of differently stained digital histopathology whole slide images. Slide-specific models for segmentation of differently stained digital histopathology whole slide images.

[48] Powers DMW (2011). Evaluation: from precision, recall and F-measure to ROC, Informedness, Markedness & Correlation. J Mach Learn Technol.

[49] Efron B (1987). Better bootstrap confidence intervals. J Am Stat Assoc.

[50] r Core Team. A language and environment for statistical computing. 2016; https://cran.r-project.org. Accessed 5 May 2017.

[51] Stevenson M, Nunes T, Heuer C, et al. epiR: tools for the analysis of epidemiological data. 2017; https://cran.r-project.org/web/packages/epiR/index.html. Accessed 5 May 2017.

[52] Warnes GR, Bolker B, Gorjanc G, et al. gdata: Various R programming tools for data manipulation. 2017; https://CRAN.R-project.org/package=gdata. Accessed 18 July 2017.

[53] Wickham H. Scales: scale functions for visualization. 2016; https://rdrr.io/cran/scales. Accessed 18 July 2017.

[54] Wickham H. ggplot2: Elegant graphics for data analysis. 2009; https://cran.r-project.org/web/packages/ggplot2/index.html. Accessed 5 May 2017.

[55] Lin LI (1989). A concordance correlation coefficient to evaluate reproducibility. Biometrics.

[56] Pearson K (1895). Note on regression and inheritance in the case of two parents. Proc R Soc London.

[57] Spearman C (1904). The proof and measurement of association between two things. Am J Psychol.

[58] McBride GB (2005). Using statistical methods for water quality management: issues, problems, and solutions.

[59] Enwere EK, Kornaga EN, Dean M (2017). Expression of PD-L1 and presence of CD8-positive T cells in pre-treatment specimens of locally advanced cervical cancer. Mod Pathol.

[60] Nowicki TS, Akiyama R, Huang RR (2017). Infiltration of CD8 T cells and expression of PD-1 and PD-L1 in synovial sarcoma. Cancer Immunol Res.

[61] Ritter AT, Asano Y, Stinchcombe JC (2015). Actin depletion initiates events leading to granule secretion at the immunological synapse. Immunity.

[62] Ohgami RS, Zhao S, Natkunam Y (2014). Large B-cell lymphomas poor in B cells and rich in PD-1+ T cells can mimic T-cell lymphomas. Am J Clin Pathol.

[63] Rizvi J, Brahmer JR, Ou SHI, et al. Safety and clinical activity of MEDI4736, an anti-programmed cell death-ligand 1 (PD-L1) antibody, in patients with non-small cell lung cancer. J Clin Oncol. 2015;33(15_suppl):8032–8032.

[64] Lesokhin AM, Ansell SM, Armand P (2016). Nivolumab in patients with relapsed or refractory hematologic malignancy: preliminary results of a phase Ib study. J Clin Oncol.

[65] Maia MC, Hansen AR (2017). A comprehensive review of immunotherapies in prostate cancer. Crit Rev Oncol Hematol.

[66] Danaher P, Warren S, Dennis L (2017). Gene expression markers of tumor infiltrating leukocytes. J Immunother Cancer.

[67] Bhat P, Leggatt G, Matthaei KI, Frazer IH (2014). The kinematics of cytotoxic lymphocytes influence their ability to kill target cells. PLoS One.

[68] Rudd CE (2008). The reverse stop-signal model for CTLA4 function. Nat Rev Immunol.

[69] Althammer S, Steele K, Rebelatto M (2016). Combinatorial CD8+ and PD-L1+ cell densities correlate with response and improved survival in non-small cell lung cancer (NSCLC) patients treated with durvalumab. J Immunother Cancer..

