# Supplementary material for: Measuring multiple parameters of CD8+ tumor-infiltrating lymphocytes in human cancers by image analysis
Source: J Immunother Cancer. 2018 Mar 6;6:20. doi: 10.1186/s40425-018-0326-x (PMC5839005; doi:10.1186/s40425-018-0326-x)
Supplement: Supplementary file 1 — Table S1 Relevant image information for nonclinical sample sets. Table S2 Nonclinical tumor samples, key patient data.Table S3 Overview of data used for statistical analysis. Table S4 Validation of CD8 IA using CD8 single stain and CD8/PD-L1 dual stain. (DOCX 91 kb) [file 40425_2018_326_MOESM1_ESM.docx]

**Measuring Multiple Parameters of CD8+ Tumor-Infiltrating Lymphocytes in Human Cancers by Image Analysis**

Steele KE, Tan TH, Korn R, Dacosta K, Brown C, Kuziora M, Zimmerman J, Laffin B, Widmaier M, Rognoni L, Cardenes R, Schneider K, Boutrin A, Martin P, Zha J, Wiestler T

**Additional file 2: Supplementary Tables**

**Table S1.** Relevant image information for nonclinical sample sets

| **Type** | **Scanned** | **Passed QC** | **Contain IM** |
| --- | --- | --- | --- |
| DLBCL | 50 | 45 | NA |
| GEC | 50 | 50 | 49 |
| LNSQ | 50 | 42 | 42 |
| LSCC | 41 | 41 | 37 |
| PANC | 50 | 48 | 27 |
| PROS | 50 | 50 | 50 |
| RCC | 50 | 49 | 41 |
| HNSCC | 50 | 50 | 34 |
| UBC | 50 | 50 | 50 |

Shown are the numbers of commercially obtained tumor specimens of each indication that were immunostained and digitally scanned, passed quality control, and contained a histologically recognizable IM that was manually annotated by a pathologist. All specimens, by definition, contained a TC. For DLBCL, the IM was not annotated to avoid uncertainty between tumor-reactive CD8+ lymphocytes and any nonreactive (resident lymphoid tissue) lymphocytes contained along the IM.

DLBCL: diffuse large B-cell lymphoma; GEC: gastroesophageal carcinoma; HNSCC: head and neck squamous-cell carcinoma; IM: invasive margin; LNSQ: nonsquamous non–small-cell lung carcinoma; LSCC: squamous-cell non–small-cell lung carcinoma; NA: not applicable; PANC: pancreatic carcinoma; PROS: prostate carcinoma; QC: quality control; RCC: renal cell carcinoma; UBC: urothelial bladder carcinoma.

**Table S2.** Nonclinical tumor samples, key patient data

| **Indication** | **Age** | **Gender** | **Ethnicity** | **Stage** | **Median Age** | **F/M/na** |
| --- | --- | --- | --- | --- | --- | --- |
| DLBCL | 55 | F | na | IV | 60.5 | 23/22/0 |
| DLBCL | 63 | M | na | IV |  |  |
| DLBCL | 69 | F | na | III |  |  |
| DLBCL | 37 | F | na | II |  |  |
| DLBCL | 70 | F | na | III |  |  |
| DLBCL | 60 | M | na | IV |  |  |
| DLBCL | 55 | F | na | II |  |  |
| DLBCL | 53 | F | na | III |  |  |
| DLBCL | 53 | M | 1 | IV |  |  |
| DLBCL | 67 | M | na | IV |  |  |
| DLBCL | 70 | F | na | IV |  |  |
| DLBCL | 61 | M | 1 | na |  |  |
| DLBCL | 55 | F | na | IV |  |  |
| DLBCL | 65 | F | na | IV |  |  |
| DLBCL | 22 | M | 2 | III |  |  |
| DLBCL | 44 | M | 1 | IVEA |  |  |
| DLBCL | 62 | M | na | IV |  |  |
| DLBCL | 48 | M | na | IV |  |  |
| DLBCL | 65 | M | 3 | IIIA |  |  |
| DLBCL | 69 | M | na | III |  |  |
| DLBCL | 27 | M | 2 | IE |  |  |
| DLBCL | 40 | F | 3 | II |  |  |
| DLBCL | 63 | M | 1 | na |  |  |
| DLBCL | 71 | F | na | na |  |  |
| DLBCL | 34 | F | 1 | na |  |  |
| DLBCL | 57 | F | 1 | II |  |  |
| DLBCL | 62 | F | 1 | IV |  |  |
| DLBCL | 54 | M | 1 | IV |  |  |
| DLBCL | 81 | F | na | IIIC |  |  |
| DLBCL | 61 | M | 1 | na |  |  |
| DLBCL | 71 | M | 1 | na |  |  |
| DLBCL | 51 | F | 1 | na |  |  |
| DLBCL | 54 | F | 1 | na |  |  |
| DLBCL | 25 | F | 1 | IA |  |  |
| DLBCL | 40 | M | 1 | na |  |  |
| DLBCL | 71 | F | 1 | IIIA |  |  |
| DLBCL | 60 | F | 1 | III |  |  |
| DLBCL | 69 | M | 1 | III |  |  |
| DLBCL | 73 | M | 1 | na |  |  |
| DLBCL | 79 | M | 1 | na |  |  |
| DLBCL | na | M | 1 | na |  |  |
| DLBCL | 58 | F | na | IV |  |  |
| DLBCL | 77 | F | na | III |  |  |
| DLBCL | 57 | F | na | III |  |  |
| DLBCL | 68 | M | na | II |  |  |
| GEC | 71 | F | 1 | T3N2M1 | 62 | 17/33/0 |
| GEC | 40 | M | 1 | T3N1M0 |  |  |
| GEC | 70 | M | 1 | T3N0M0 |  |  |
| GEC | 69 | M | 1 | T3N3M0 |  |  |
| GEC | 73 | M | 1 | T3N2M0 |  |  |
| GEC | 51 | M | 1 | T3N3aM0 |  |  |
| GEC | 69 | F | 1 | T3N1M0 |  |  |
| GEC | 52 | F | na | T1N1M1 |  |  |
| GEC | 49 | M | 1 | T3N0M0 |  |  |
| GEC | 55 | M | 1 | T3N1M0 |  |  |
| GEC | 64 | M | 1 | T3N2M0 |  |  |
| GEC | 53 | M | 1 | T3N2M0 |  |  |
| GEC | 62 | F | 1 | T3N0M0 |  |  |
| GEC | 52 | M | 1 | T4bN0M0 |  |  |
| GEC | 71 | M | 1 | T3N3aM1 |  |  |
| GEC | 62 | F | 1 | T3N0M0 |  |  |
| GEC | 41 | F | na | T4N0M0 |  |  |
| GEC | 68 | M | 1 | T3N0M0 |  |  |
| GEC | 56 | M | na | T3N0MX |  |  |
| GEC | 72 | M | na | T3N0MX |  |  |
| GEC | 70 | M | 1 | T3N2M0 |  |  |
| GEC | 62 | M | 1 | T3N3aM0 |  |  |
| GEC | 62 | M | 1 | T3N1M0 |  |  |
| GEC | 59 | F | 1 | T2N0M0 |  |  |
| GEC | 71 | F | 1 | T3N2M0 |  |  |
| GEC | 54 | M | 1 | T3N0M0 |  |  |
| GEC | 65 | M | 1 | T3N3M0 |  |  |
| GEC | 70 | F | 2 | T3N0M0 |  |  |
| GEC | 75 | F | 1 | T3N1M0 |  |  |
| GEC | 57 | M | 1 | T3N3bM0 |  |  |
| GEC | 49 | M | 1 | T3N0M0 |  |  |
| GEC | 55 | M | 1 | T2N1M0 |  |  |
| GEC | 64 | F | 1 | T3N1M0 |  |  |
| GEC | 61 | M | 1 | T3N3M0 |  |  |
| GEC | 45 | M | 1 | T3N3aM0 |  |  |
| GEC | 62 | F | 1 | T3N1M0 |  |  |
| GEC | 55 | M | 1 | T3N1M0 |  |  |
| GEC | 59 | M | 1 | NT3N2M0 |  |  |
| GEC | 64 | M | 1 | T3N2M0 |  |  |
| GEC | 72 | F | 1 | T4bN0M0 |  |  |
| GEC | 63 | M | 1 | T3N1M0 |  |  |
| GEC | 66 | M | 1 | T3N2M0 |  |  |
| GEC | 59 | F | 1 | T3N3M0 |  |  |
| GEC | 73 | F | 1 | T3N2M0 |  |  |
| GEC | 51 | M | 1 | T1bN0M0 |  |  |
| GEC | 80 | M | 1 | T3N0M0 |  |  |
| GEC | 57 | M | 1 | T3N1M0 |  |  |
| GEC | 63 | F | 1 | T4bN2M0 |  |  |
| GEC | 72 | F | 1 | T3N0M0 |  |  |
| GEC | 62 | M | 1 | T3N2M1 |  |  |
| LNSQ | 49 | na | na | (IA) | 60 | 15/26/1 |
| LNSQ | 62 | M | 3 | T2aNXM0 |  |  |
| LNSQ | 67 | F | na | T1aN0MX |  |  |
| LNSQ | 77 | F | 1 | na |  |  |
| LNSQ | 61 | M | 3 | na |  |  |
| LNSQ | 69 | F | 3 | T2aN1M0 |  |  |
| LNSQ | 55 | M | 3 | T2aN0M0 |  |  |
| LNSQ | 49 | M | 3 | T3N0M0 |  |  |
| LNSQ | 69 | M | 3 | T1bN0M0 |  |  |
| LNSQ | 63 | F | 1 | T2bN2M0 |  |  |
| LNSQ | 58 | M | 3 | T1bN0M0 |  |  |
| LNSQ | 62 | M | 1 | T2aN1M0 |  |  |
| LNSQ | 49 | M | 3 | T2aNXM0 |  |  |
| LNSQ | 59 | M | na | (IIB) |  |  |
| LNSQ | 66 | F | 1 | T2aN1M0 |  |  |
| LNSQ | 64 | M | 1 | T2bN0M0 |  |  |
| LNSQ | 75 | M | 1 | T1bN0M0 |  |  |
| LNSQ | 52 | F | na | (IIA) |  |  |
| LNSQ | 67 | M | na | (IIIA) |  |  |
| LNSQ | 72 | F | 1 | T1bN0M0 |  |  |
| LNSQ | 53 | F | 1 | T1aN0M0 |  |  |
| LNSQ | 45 | M | 1 | T2bN0M0 |  |  |
| LNSQ | 50 | F | 3 | T2aN0M0 |  |  |
| LNSQ | 60 | F | 1 | T1bN0M0 |  |  |
| LNSQ | 54 | M | 3 | T2aN0M0 |  |  |
| LNSQ | 64 | M | 1 | T2aN0M0 |  |  |
| LNSQ | 53 | M | 1 | T2aN0M0 |  |  |
| LNSQ | 56 | M | 1 | T3N0M0 |  |  |
| LNSQ | 56 | F | 1 | T1aN0M0 |  |  |
| LNSQ | 54 | F | 1 | T2aN2M0 |  |  |
| LNSQ | 53 | M | 3 | T1bN0MX |  |  |
| LNSQ | 58 | F | 3 | T2aN0M0 |  |  |
| LNSQ | 68 | M | 1 | T1bN0M0 |  |  |
| LNSQ | 64 | M | 1 | T2bN1M0 |  |  |
| LNSQ | 57 | F | na | (IB) |  |  |
| LNSQ | 68 | F | na | (IIB) |  |  |
| LNSQ | 65 | M | na | T2aN0MX |  |  |
| LNSQ | 57 | M | 1 | T2bN2M0 |  |  |
| LNSQ | 60 | M | na | (IB) |  |  |
| LNSQ | 58 | M | na | (IIIA) |  |  |
| LNSQ | 62 | M | 1 | T2bN2M0 |  |  |
| LNSQ | 67 | M | 1 | T1bN0M0 |  |  |
| LSCC | 52 | F | na | na | 58 | 4/37/0 |
| LSCC | 56 | M | na | T2bN0M0 |  |  |
| LSCC | 73 | M | na | T1N0M0 |  |  |
| LSCC | 70 | M | na | T2aN0M0 |  |  |
| LSCC | 62 | M | na | T2aN2M0 |  |  |
| LSCC | 56 | M | na | T2bNXM0 |  |  |
| LSCC | 60 | F | 1 | T2N0M0 |  |  |
| LSCC | 63 | M | na | T2N0M0 |  |  |
| LSCC | 52 | M | 1 | T3N0M0 |  |  |
| LSCC | 62 | M | na | T2N0M0 |  |  |
| LSCC | 69 | M | na | T2bN0M0 |  |  |
| LSCC | 74 | M | na | T2N0M0 |  |  |
| LSCC | 53 | F | 1 | T2N0M0 |  |  |
| LSCC | 63 | M | na | T3N0M0 |  |  |
| LSCC | 52 | M | na | T3N0M0 |  |  |
| LSCC | 58 | M | na | T2aN0M0 |  |  |
| LSCC | 59 | M | na | T3N2M0 |  |  |
| LSCC | 55 | M | na | T2N1M0 |  |  |
| LSCC | 60 | M | na | T1N1M0 |  |  |
| LSCC | 62 | M | 1 | na |  |  |
| LSCC | 74 | M | 1 | na |  |  |
| LSCC | 65 | F | na | T1N0M0 |  |  |
| LSCC | 59 | M | 1 | T2N0M0 |  |  |
| LSCC | 54 | M | na | na |  |  |
| LSCC | 59 | M | na | T2aN1M0 |  |  |
| LSCC | 61 | M | na | T2N0M0 |  |  |
| LSCC | 58 | M | na | T2N0MX |  |  |
| LSCC | 69 | M | 1 | T3N0M0 |  |  |
| LSCC | 61 | M | na | T2N0M0 |  |  |
| LSCC | 75 | M | na | T2N0M0 |  |  |
| LSCC | 73 | M | 1 | na |  |  |
| LSCC | 72 | M | 1 | T2N0MX |  |  |
| LSCC | 59 | M | 1 | na |  |  |
| LSCC | 56 | M | na | T2bN0M0 |  |  |
| LSCC | 68 | M | 1 | T2NXM0 |  |  |
| LSCC | 71 | M | na | T2bNXM0 |  |  |
| LSCC | 66 | M | na | T1bNXM0 |  |  |
| LSCC | 65 | M | na | T2N1M0 |  |  |
| LSCC | 63 | M | 1 | T2aN0M0 |  |  |
| LSCC | 74 | M | na | T1N0M0 |  |  |
| LSCC | 50 | M | na | T2N1M0 |  |  |
| PANC | na | na | na | na | 64.5 | 26/16/6 |
| PANC | 59 | M | 1 | T3N1M0 |  |  |
| PANC | 63 | F | 1 | T4N0M0 |  |  |
| PANC | na | na | na | na |  |  |
| PANC | 63 | F | na | T3NXM0 |  |  |
| PANC | 60 | F | 1 | T4N0M0 |  |  |
| PANC | 58 | F | 1 | T2N0M0 |  |  |
| PANC | 83 | F | 1 | T3N1MX |  |  |
| PANC | 73 | M | na | na |  |  |
| PANC | 54 | F | 1 | T3N1M0 |  |  |
| PANC | 56 | F | 1 | T3N0M0 |  |  |
| PANC | 25 | F | na | na |  |  |
| PANC | 81 | M | na | na |  |  |
| PANC | 68 | M | na | na |  |  |
| PANC | 60 | F | na | na |  |  |
| PANC | 59 | F | 1 | T3N1M0 |  |  |
| PANC | 65 | F | na | na |  |  |
| PANC | 78 | F | na | na |  |  |
| PANC | 77 | M | 1 | T1N0M0 |  |  |
| PANC | 54 | M | 1 | T3N1M0 |  |  |
| PANC | 74 | F | 1 | T3N1M0 |  |  |
| PANC | 53 | M | na | T3N0M0 |  |  |
| PANC | 57 | F | 1 | T3N1M0 |  |  |
| PANC | 61 | F | 1 | T3N0M0 |  |  |
| PANC | 48 | M | 1 | T4N0M0 |  |  |
| PANC | 71 | F | na | na |  |  |
| PANC | 71 | F | 1 | T3N0M0 |  |  |
| PANC | 66 | M | 1 | T3N1M0 |  |  |
| PANC | na | na | na | na |  |  |
| PANC | 72 | F | 1 | T3N0M0 |  |  |
| PANC | 68 | M | na | na |  |  |
| PANC | 60 | M | 1 | T3N1M0 |  |  |
| PANC | 57 | F | 1 | T3N1M0 |  |  |
| PANC | 56 | M | 1 | T3N1M0 |  |  |
| PANC | na | na | na | na |  |  |
| PANC | 79 | M | 1 | T3N0M0 |  |  |
| PANC | 68 | F | 1 | T2NXM0 |  |  |
| PANC | 69 | M | na | na |  |  |
| PANC | 64 | F | 1 | T3N0M0 |  |  |
| PANC | 75 | F | 1 | T3N0M0 |  |  |
| PANC | 65 | F | 1 | T3N0M0 |  |  |
| PANC | 67 | M | na | na |  |  |
| PANC | 74 | M | na | na |  |  |
| PANC | 73 | F | 1 | T3N0MX |  |  |
| PANC | na | na | na | na |  |  |
| PANC | na | na | na | na |  |  |
| PANC | 62 | F | 1 | T3N0M0 |  |  |
| PANC | 62 | F | 1 | T3N0M0 |  |  |
| PROS | 57 | M | 1 | T2cN0M0 | 62.5 | 0/50/0 |
| PROS | 64 | M | 1 | T3bN0M0 |  |  |
| PROS | 66 | M | na | T3NXMX |  |  |
| PROS | 68 | M | na | T3bN0MX |  |  |
| PROS | 47 | M | na | T3N0MX |  |  |
| PROS | 53 | M | na | T3aN0MX |  |  |
| PROS | 71 | M | na | T3bNXMX |  |  |
| PROS | 55 | M | na | T3aNXMX |  |  |
| PROS | 53 | M | na | T3aN0MX |  |  |
| PROS | 63 | M | 1 | T2cN0M0 |  |  |
| PROS | 66 | M | na | T3aNXMX |  |  |
| PROS | 54 | M | 1 | T3bN0M0 |  |  |
| PROS | 66 | M | 1 | T3aN0M0 |  |  |
| PROS | 60 | M | 1 | T2cN0M0 |  |  |
| PROS | 43 | M | na | T3N0MX |  |  |
| PROS | 67 | M | 1 | T3bN1M0 |  |  |
| PROS | 63 | M | 1 | T3aN0M0 |  |  |
| PROS | 59 | M | 1 | T2cN0M0 |  |  |
| PROS | 59 | M | 1 | T2cN0M0 |  |  |
| PROS | 64 | M | 1 | T2cN0M0 |  |  |
| PROS | 63 | M | 1 | T3aN0M0 |  |  |
| PROS | 60 | M | 1 | T3aN0M0 |  |  |
| PROS | 62 | M | 1 | T2cN0M0 |  |  |
| PROS | 63 | M | 1 | T3aN0M0 |  |  |
| PROS | 59 | M | 1 | T3bN0M0 |  |  |
| PROS | 72 | M | na | T3N0MX |  |  |
| PROS | 51 | M | na | T3aNXMX |  |  |
| PROS | 62 | M | 1 | T2cN0M0 |  |  |
| PROS | 67 | M | 1 | T3bN0M0 |  |  |
| PROS | 67 | M | 1 | T2cN0M0 |  |  |
| PROS | 49 | M | 1 | T3aN0M0 |  |  |
| PROS | 71 | M | 1 | T3bN0M0 |  |  |
| PROS | 64 | M | 1 | T2cN1M0 |  |  |
| PROS | 67 | M | 1 | T3aN0M0 |  |  |
| PROS | 61 | M | 1 | T3bN0M0 |  |  |
| PROS | 69 | M | 1 | T3aN1M0 |  |  |
| PROS | 64 | M | 1 | T3bN0M0 |  |  |
| PROS | 55 | M | 1 | T2cN0M0 |  |  |
| PROS | 62 | M | 1 | T3aN0M0 |  |  |
| PROS | 52 | M | 1 | T2cN0M0 |  |  |
| PROS | 60 | M | 1 | T2cN0M0 |  |  |
| PROS | 67 | M | 1 | T2cN0M0 |  |  |
| PROS | 70 | M | 1 | T2cN0M0 |  |  |
| PROS | 65 | M | 1 | T2bN0M0 |  |  |
| PROS | 62 | M | 1 | T3bN0M0 |  |  |
| PROS | 51 | M | 1 | T2cN0M0 |  |  |
| PROS | 67 | M | 1 | T3aN0M0 |  |  |
| PROS | 63 | M | 1 | T3bN0M0 |  |  |
| PROS | 53 | M | 1 | T3bN0M0 |  |  |
| PROS | 65 | M | 1 | T2cN0M0 |  |  |
| RCC | 60 | M | 1 | T3aNXM0 | 58.5 | 9/12/28 |
| RCC | 74 | na | na | T3aN1MX |  |  |
| RCC | 39 | na | na | T1bN0MX |  |  |
| RCC | na | na | na | na |  |  |
| RCC | na | M | 1 | T1aNXMX |  |  |
| RCC | 73 | na | na | T3aNXMX |  |  |
| RCC | na | na | na | na |  |  |
| RCC | 71 | na | na | T1bNXMX |  |  |
| RCC | 51 | na | na | T3aNXMX |  |  |
| RCC | 48 | M | 1 | T1aNXMX |  |  |
| RCC | 66 | F | 1 | T2NXM0 |  |  |
| RCC | 48 | M | 1 | T1bNXM0 |  |  |
| RCC | 64 | F | 1 | T1bN0M0 |  |  |
| RCC | 60 | na | na | T3aN0MX |  |  |
| RCC | 69 | M | 1 | T2N0M0 |  |  |
| RCC | 66 | M | 1 | T1bNXM0 |  |  |
| RCC | 33 | na | na | T3aNXMX |  |  |
| RCC | 40 | na | na | T1aNXMX |  |  |
| RCC | 55 | na | na | T1bNXMX |  |  |
| RCC | 64 | M | 1 | T1bNXM0 |  |  |
| RCC | 60 | na | na | T3aNXMX |  |  |
| RCC | 75 | na | na | T3aN2MX |  |  |
| RCC | 56 | na | na | T3aNXMX |  |  |
| RCC | 75 | M | 1 | T3N0M0 |  |  |
| RCC | 55 | F | 1 | T3N2MX |  |  |
| RCC | 56 | na | na | T2bNXMX |  |  |
| RCC | 68 | M | 1 | T1aNXMX |  |  |
| RCC | 55 | na | na | T1bNXMX |  |  |
| RCC | 39 | na | na | T3aNXMX |  |  |
| RCC | 64 | na | na | T3aNXMX |  |  |
| RCC | 70 | F | 1 | T1bNXMX |  |  |
| RCC | 66 | na | na | T3aNXMX |  |  |
| RCC | 61 | na | na | T1bNXMX |  |  |
| RCC | 55 | na | na | T2bNXMX |  |  |
| RCC | 54 | na | na | T2aNXMX |  |  |
| RCC | 65 | M | 1 | T1bNXM0 |  |  |
| RCC | 45 | M | 1 | T1NXM0 |  |  |
| RCC | 64 | F | 1 | T1bNXMX |  |  |
| RCC | 57 | M | 1 | T3aN0M0 |  |  |
| RCC | 43 | na | na | T1aNXMX |  |  |
| RCC | 54 | na | na | T1bNXMX |  |  |
| RCC | 56 | na | na | T3aNXMX |  |  |
| RCC | 61 | na | na | T3aNXMX |  |  |
| RCC | 50 | F | 1 | T1N0M0 |  |  |
| RCC | 46 | F | 1 | T2N0M0 |  |  |
| RCC | 80 | F | 1 | T2N0M0 |  |  |
| RCC | 68 | F | 1 | T2NXM0 |  |  |
| RCC | 39 | na | na | T1aNXMX |  |  |
| RCC | 54 | na | na | T1bNXMX |  |  |
| HNSCC | 58 | M | na | T4aNXMX | 56.5 | 12/38/0 |
| HNSCC | 54 | M | 1 | T2N0M0 |  |  |
| HNSCC | na | M | 1 | T1N2bM0 |  |  |
| HNSCC | 59 | M | 1 | T3N1M0 |  |  |
| HNSCC | na | M | 1 | T4aN2bM0 |  |  |
| HNSCC | 46 | M | 1 | T3N0M0 |  |  |
| HNSCC | 62 | F | 1 | T2N0M0 |  |  |
| HNSCC | na | M | 1 | T3N1M0 |  |  |
| HNSCC | 46 | M | 1 | T3N0M0 |  |  |
| HNSCC | 76 | M | 1 | T4aN0M0 |  |  |
| HNSCC | na | M | 1 | T3N0M0 |  |  |
| HNSCC | na | F | 1 | T3N0M0 |  |  |
| HNSCC | na | F | 1 | T3N1M0 |  |  |
| HNSCC | na | M | 1 | na |  |  |
| HNSCC | na | M | 1 | T4N1M0 |  |  |
| HNSCC | 74 | F | 1 | T4aN0M0 |  |  |
| HNSCC | na | M | 1 | T4aN2cMX |  |  |
| HNSCC | na | M | 1 | T2N2M0 |  |  |
| HNSCC | na | M | 1 | T3N0M0 |  |  |
| HNSCC | na | M | 1 | T2N2bM0 |  |  |
| HNSCC | 55 | M | 1 | T3N0M0 |  |  |
| HNSCC | na | M | 1 | T4aN2cM0 |  |  |
| HNSCC | na | F | 1 | T3N1M0 |  |  |
| HNSCC | 64 | M | 1 | T4aN0M0 |  |  |
| HNSCC | na | M | 1 | T2N1M0 |  |  |
| HNSCC | 63 | M | 1 | T3N0M0 |  |  |
| HNSCC | na | M | 2 | T1N1M0 |  |  |
| HNSCC | na | M | 1 | T4aN0M0 |  |  |
| HNSCC | na | M | 1 | T3N0M0 |  |  |
| HNSCC | na | M | 1 | T4aN1MX |  |  |
| HNSCC | 73 | M | 1 | T2N0M0 |  |  |
| HNSCC | na | m | 1 | T1N2cM0 |  |  |
| HNSCC | na | M | 1 | T1N2bM0 |  |  |
| HNSCC | 57 | M | 1 | T4aN0M0 |  |  |
| HNSCC | 45 | M | 1 | T3N2bM0 |  |  |
| HNSCC | 42 | F | 1 | T4aN0M0 |  |  |
| HNSCC | 62 | M | 1 | T4aN0M0 |  |  |
| HNSCC | na | M | 1 | T4aN0M0 |  |  |
| HNSCC | 56 | M | 1 | T4aN0M0 |  |  |
| HNSCC | 63 | F | 1 | T3N0M0 |  |  |
| HNSCC | 50 | F | 1 | T3N2bM0 |  |  |
| HNSCC | 63 | M | 1 | T4aN2bM0 |  |  |
| HNSCC | na | M | 1 | T4N0M0 |  |  |
| HNSCC | 47 | F | 1 | T3N1M0 |  |  |
| HNSCC | na | M | 1 | T4aN2bM0 |  |  |
| HNSCC | 50 | F | 1 | T4aN1M0 |  |  |
| HNSCC | 31 | F | 1 | T3N2bM0 |  |  |
| HNSCC | 38 | M | 1 | T4aN2bM0 |  |  |
| HNSCC | 89 | F | 1 | T2N0M0 |  |  |
| HNSCC | 51 | M | 1 | T3N0M0 |  |  |
| UBC | 75 | M | 1 | TXN0M0 | 71 | 6/44/0 |
| UBC | 64 | M | na | T4NXMX |  |  |
| UBC | 74 | M | 1 | T4aN1MX |  |  |
| UBC | 67 | M | na | T2NXMX |  |  |
| UBC | 53 | M | 1 | T2bN0M0 |  |  |
| UBC | 77 | M | na | na |  |  |
| UBC | 77 | M | na | T3aN0MX |  |  |
| UBC | 47 | M | 1 | T4N2M0 |  |  |
| UBC | 74 | M | na | T1NXMX |  |  |
| UBC | 79 | M | na | T3aNXMX |  |  |
| UBC | 73 | F | na | T3NXMX |  |  |
| UBC | 56 | M | 1 | T4N2M0 |  |  |
| UBC | 64 | M | 1 | T2bN0M0 |  |  |
| UBC | 60 | M | 1 | T4N0M0 |  |  |
| UBC | 76 | M | na | T3aNXMX |  |  |
| UBC | 70 | M | 1 | T4aN0MX |  |  |
| UBC | 61 | M | 1 | T2bN0M0 |  |  |
| UBC | 54 | M | na | T2NXMX |  |  |
| UBC | 76 | F | na | TaNXMX |  |  |
| UBC | 72 | M | na | T1NXMX |  |  |
| UBC | 83 | M | na | T1NXMX |  |  |
| UBC | 73 | M | na | T1NXMX |  |  |
| UBC | 85 | M | na | T1NXMX |  |  |
| UBC | 72 | F | na | T2NXMX |  |  |
| UBC | 67 | M | na | T1aNXMX |  |  |
| UBC | 92 | F | na | T2NXMX |  |  |
| UBC | 72 | M | na | T4aN0MX |  |  |
| UBC | 79 | M | na | T3bN0MX |  |  |
| UBC | 70 | M | 1 | T2bN0M0 |  |  |
| UBC | 60 | M | na | T3aN0MX |  |  |
| UBC | 63 | M | na | T3NXMX |  |  |
| UBC | 73 | F | na | TaNXMX |  |  |
| UBC | 65 | M | 1 | T3N2MX |  |  |
| UBC | 77 | M | na | T4NXMX |  |  |
| UBC | 91 | M | na | T3aNXMX |  |  |
| UBC | 48 | M | 1 | T2N0MX |  |  |
| UBC | 67 | M | 1 | TXN0MX |  |  |
| UBC | 72 | M | 1 | T2bN0M0 |  |  |
| UBC | 68 | M | 1 | T4aN1M0 |  |  |
| UBC | 60 | F | na | T2NXMX |  |  |
| UBC | 66 | M | na | T2NXMX |  |  |
| UBC | 71 | M | 1 | T4NXMX |  |  |
| UBC | 55 | M | 1 | T3N0MX |  |  |
| UBC | na | M | 1 | T3bN0MX |  |  |
| UBC | 88 | M | na | T2NXMX |  |  |
| UBC | 69 | M | na | T4aNXMX |  |  |
| UBC | 81 | M | na | T2aNXMX |  |  |
| UBC | 63 | M | 1 | T3N1M0 |  |  |
| UBC | 84 | M | na | TXNXMX |  |  |
| UBC | 71 | M | na | T1NXMX |  |  |

Shown is the key patient-related information provided by the vendor for each nonclinical specimen analyzed.

DLBCL: diffuse large B-cell lymphoma; F: female; GEC: gastroesophageal carcinoma; HNSCC: head and neck squamous-cell carcinoma; LNSQ: non-squamous non–small-cell lung carcinoma; LSCC: squamous-cell non–small-cell lung carcinoma; M: male; na: not available; PANC: pancreatic carcinoma; PROS: prostate carcinoma; RCC: renal cell carcinoma; UBC: urothelial bladder carcinoma. Ethnicity codes: 1, caucasian or white/non-Hispanic; 2, black or African-American; 3, Asian/Pacific Islander.

**Table S3.** Overview of data used for statistical analysis

| **Sample set** | **Comparison** | **FOVs** | **P** | **IA** | **FN** | **FP** | **TP** |
| --- | --- | --- | --- | --- | --- | --- | --- |
| DLBCL | IA-P1 | 20 | 4477 | 5752 | 405 | 1680 | 4072 |
| GEC | IA-P1 | 20 | 1740 | 1835 | 437 | 532 | 1303 |
| LNSQ | IA-P1 | 18 | 1273 | 1753 | 181 | 661 | 1092 |
| LSCC | IA-P1 | 20 | 1795 | 2164 | 408 | 777 | 1387 |
| PANC | IA-P1 | 18 | 3685 | 4854 | 410 | 1579 | 3275 |
| PROS | IA-P1 | 20 | 1272 | 1744 | 120 | 592 | 1152 |
| RCC | IA-P1 | 20 | 822 | 1082 | 107 | 367 | 715 |
| HNSCC | IA-P1 | 21 | 1366 | 1167 | 342 | 143 | 1024 |
| UBC | IA-P1 | 22 | 833 | 1255 | 58 | 480 | 775 |
| NCT01693562_MonoStain_1P | IA-P1 | 51 | 2800 | 2717 | 1128 | 1045 | 1672 |
| NCT01693562_MonoStain_3P | IA-PC | 13 | 1744 | 2181 | 143 | 580 | 1601 |
| NCT01693562_MonoStain_3P | IA-P1 | 13 | 1820 | 2435 | 171 | 786 | 1649 |
| NCT01693562_MonoStain_3P | IA-P2 | 13 | 1661 | 2602 | 142 | 1083 | 1519 |
| NCT01693562_MonoStain_3P | IA-P3 | 13 | 2131 | 2651 | 325 | 845 | 1806 |
| NCT01693562_MonoStain_3P | P1-P2 | 13 | 1662 | 1800 | 227 | 365 | 1435 |
| NCT01693562_MonoStain_3P | P1-P3 | 13 | 2131 | 1825 | 554 | 248 | 1577 |
| NCT01693562_MonoStain_3P | P2-P1 | 13 | 1825 | 1562 | 390 | 127 | 1435 |
| NCT01693562_MonoStain_3P | P2-P3 | 13 | 2131 | 1662 | 674 | 205 | 1457 |
| NCT01693562_MonoStain_3P | P3-P1 | 13 | 1825 | 1962 | 249 | 386 | 1576 |
| NCT01693562_MonoStain_3P | P3-P2 | 13 | 1662 | 2093 | 204 | 635 | 1458 |
| NCT01693562_DualStain_2P | IA-PC | 18 | 957 | 1448 | 146 | 637 | 811 |
| NCT01693562_DualStain_2P | IA-P1 | 18 | 1044 | 1840 | 168 | 964 | 876 |
| NCT01693562_DualStain_2P | IA-P2 | 18 | 1519 | 1840 | 381 | 702 | 1138 |
| NCT01693562_DualStain_2P | P1-P2 | 18 | 1519 | 1045 | 562 | 88 | 957 |
| NCT01693562_DualStain_2P | P2-P1 | 18 | 1045 | 1519 | 88 | 562 | 957 |
| NCT01693562_MonoStain_2P | IA-PC | 18 | 1259 | 1714 | 156 | 611 | 1103 |
| NCT01693562_MonoStain_2P | IA-P1 | 18 | 1409 | 2029 | 198 | 818 | 1211 |
| NCT01693562_MonoStain_2P | IA-P2 | 18 | 1640 | 2029 | 334 | 723 | 1306 |
| NCT01693562_MonoStain_2P | P1-P2 | 18 | 1640 | 1409 | 382 | 151 | 1258 |
| NCT01693562_MonoStain_2P | P2-P1 | 18 | 1409 | 1640 | 150 | 381 | 1259 |

Data are shown for each combination of sample set–assessed comparison. IA was compared with pathologist 1, pathologist 2, pathologist 3, and their consolidated annotations. In addition, the pathologists’ annotations were compared with each other. For each of these combinations, the sum of all counts of CD8+ TIL annotations by the respective pathologist, the IA, and the true positives, false positives, and false negatives across all FOVs, is provided.

DLBCL: diffuse large B-cell lymphoma; FN: false negative; FOV: high-magnification field of view; FP: false positive; GEC: gastroesophageal carcinoma; HNSCC: head and neck squamous-cell carcinoma; IA: image analysis; LNSQ: non-squamous non–small-cell lung carcinoma; LSCC: squamous-cell non–small-cell lung carcinoma; P: pathologist; PANC: pancreatic carcinoma; PC: pathologists, consolidated; PROS: prostate carcinoma; RCC: renal cell carcinoma; TP: true positive; UBC: urothelial bladder carcinoma.

**Table S4.** Validation of CD8 IA using CD8 single stain and CD8/PD-L1 dual stain

| **Stain** | **Comparison** | **CCC** | **CCC_lower** | **F_1_** | **F_1__lower** | **PCC** | **PCC_lower** | **SCC** | **SCC_lower** |
| --- | --- | --- | --- | --- | --- | --- | --- | --- | --- |
| Single | IA-PC | 0.81 | 0.77 | 0.74 | 0.73 | 0.97 | 0.91 | 0.93 | 0.78 |
|  | IA-P1 | 0.82 | 0.73 | 0.70 | 0.69 | 0.99 | 0.94 | 0.94 | 0.82 |
|  | IA-P2 | 0.84 | 0.81 | 0.71 | 0.70 | 0.94 | 0.86 | 0.87 | 0.68 |
|  | P1-P2 | 0.96 | 0.82 | 0.83 | 0.81 | 0.98 | 0.94 | 0.94 | 0.83 |
|  | P2-P1 | 0.96 | 0.82 | 0.83 | 0.81 | 0.98 | 0.94 | 0.94 | 0.82 |
| Dual | IA-PC | 0.73 | 0.63 | 0.67 | 0.66 | 0.96 | 0.91 | 0.91 | 0.73 |
|  | IA-P1 | 0.66 | 0.46 | 0.61 | 0.59 | 0.96 | 0.87 | 0.88 | 0.65 |
|  | IA-P2 | 0.89 | 0.83 | 0.68 | 0.66 | 0.97 | 0.92 | 0.95 | 0.86 |
|  | P1-P2 | 0.85 | 0.67 | 0.75 | 0.73 | 0.99 | 0.96 | 0.93 | 0.76 |
|  | P2-P1 | 0.85 | 0.67 | 0.75 | 0.73 | 0.99 | 0.96 | 0.93 | 0.76 |

CD8+ lymphocytes were enumerated by IA and pathologists for single-stained and double-stained NSCLC specimens, as described in the main text. Shown are the concordance values for IA compared with two pathologists and their consolidated annotations, as well as inter-pathologist comparisons. The table shows the correlation coefficients comparing counts per high-magnification field of view. In addition, agreement on a cell-by-cell basis is shown by F_1_ score. For all values, lower one-sided 95% confidence intervals are shown.
