# Supplementary material for: Measuring multiple parameters of CD8+ tumor-infiltrating lymphocytes in human cancers by image analysis
Source: J Immunother Cancer. 2018 Mar 6;6:20. doi: 10.1186/s40425-018-0326-x (PMC5839005; doi:10.1186/s40425-018-0326-x)
Supplement: Supplementary file 2 — CD8/PD-L1 dual IHC, quality control; co-registration; details of the automated classification assessment; details of digital IA scoring solutions; measurement of CD8+ TILs in PD-L1–positive tumor. (DOCX 45 kb) [file 40425_2018_326_MOESM2_ESM.docx]

**Measuring Multiple Parameters of CD8+ Tumor-Infiltrating Lymphocytes in Human Cancers by Image Analysis**

Steele KE, Tan TH, Korn R, Dacosta K, Brown C, Kuziora M, Zimmerman J, Laffin B, Widmaier M, Rognoni L, Cardenes R, Schneider K, Boutrin A, Martin P, Zha J, Wiestler T

**Supplementary Materials and Methods**

**CD8/PD-L1 dual IHC**

For the CD8/PD-L1 double stain, the Ventana Discovery Ultra instrument (Roche Diagnostics, Ventana Medical Systems, Tucson, AZ) was used. After antigen retrieval using the instrument’s cell conditioning protocol, PD-L1 antibody SP263 (Spring Bioscience, Pleasanton, CA) was detected with anti-rabbit hydroquinone (HQ) and anti–HQ-horseradish peroxidase (HRP) (catalog nos. 760-4815, 760-4820; Roche Diagnostics) combined with 3,3ʹ-diaminobenzidine (catalog no. 760-159; Roche Diagnostics). After antibody denaturation, the CD8 antibody SP239 was applied and detected with Discovery OmniMap Anti-Rabbit HRP (catalog no. 760-4311; Roche Diagnostics) combined with the Discovery purple substrate kit. Sections of human tumor known to contain abundant CD8+ TILs were used as positive control for the CD8 IHC. In addition, a monoclonal rabbit immunoglobulin G isotype antibody (catalog no. 760-1029; Roche Diagnostics) was applied to these samples as a negative reagent control. After immunostaining, nuclear counterstaining was performed and then the slides were rinsed and dehydrated and coverslips were applied with a permanent mounting medium.

**Quality control**

We assessed the results of key processes in our overall system as follows. To determine the quality of formalin-fixed, paraffin-embedded specimens, a pathologist microscopically evaluated tumor sections stained with hematoxylin and eosin. This evaluation included an assessment of the general histological preservation of tissues, the presence of sufficient amounts of viable tumor cells, and for nonclinical tumors, the presence of tumor IM. Evaluation of IHC quality included pathologist assessment of assay controls and the immunostaining results for individual slides (100% of clinical trial specimens and 20% or more of the nonclinical specimens of each indication). Scanning quality was assessed by visual inspection for sample acquisition accuracy, focus, the presence of box artifacts, and the absence of obstructive artifacts. Slides were rescanned if scanning quality was insufficient. Next, an IA scientist visually assessed 100% of the slides for preparation, staining, and the quality of scanning output required for IA. For this, IA tools were used to identify regions of concern, such as blurred areas or box artifacts. Images not meeting our quality criteria were excluded from further analysis. The numbers of samples that were considered of suitable quality for data production by IA are shown in Table S1.

**Co-registration**

The CD8 analyses performed on nonclinical samples included in this report were part of multiple-marker studies. Thus, tissue sections that were relatively serial to those in CD8-related images were also immunostained for various additional IHC markers and with hematoxylin and eosin. For each specimen, sets of serial sections were automatically co-registered digitally as previously described [1,2], and the manual annotations of tumor regions were transferred to the applicable CD8 images (main text, Fig. 1C). For co-registration of IA slides, semantic information such as tissue region was first extracted from serial slides at low resolution based on color and contrast. A global intensity-based rigid registration was then used to pre-align tissue pieces from different sections. This process was refined by iterative and hierarchical local registration, in which rigid point correspondences between sections were identified. For registration, the normalized cross-correlation on normalized intensity maps was used. The image normalization established a visual similarity between differently stained sections, leading to a more robust analysis [3]. The co-registered annotations were ultimately inspected and, when necessary (approximately 5-10% of instances), manually corrected by a pathologist to ensure accuracy of the transfers.

**Details of the automated classification assessment**

An automated classification assessment system was used to assess the quality of CD8+ TIL classification and counts, as described in the main text. For each indication, multiple high-magnification fields of view (FOV) were selected using VeriTrova, and the pathologist manually annotated each CD8+ TIL within FOVs. The software then compared each cell detected by IA with the pathologist’s annotation (or lack thereof) or compared each annotated cell detected as CD8+ by IA. To avoid incorrect assessment of cells at the border of the FOVs, the FOV outline was automatically corrected to either completely exclude or completely include any relevant marginal cells. Cell marker annotations were acquired for every CD8+ TIL within selected FOVs by either one pathologist, in the case of nonclinical specimen sets, or by two or three independent pathologists, in the case of matched biopsy pairs from clinical trial NCT01693562. The pathologist had the option to create annotations representing equivocal positivity for ambiguous findings, eg, low-level staining of fragments of cells. IA results that included only equivocal annotations were excluded from the assessment. When annotations from several pathologists existed, a set of consolidated annotations was automatically created. IA results that included an annotation in which fewer than two pathologists agreed were also excluded from the assessment. Developer XD software (Definiens), in-house software, and R, an open-source software environment for statistical computing and graphics, were used to compare annotations against CD8+ TILs classified by IA [4-7]. CD8+ TILs were categorized as false positive if they were classified by IA but not annotated, false negative if they were annotated but not classified by IA, and true positive if IA and annotation agreed. Based on this classification, CCC, PCC, SCC, and F_1_ score were calculated.

**Details of digital IA scoring solutions**

We employed a machine learning approach for the detection of negative nuclei by detecting prototypical hematoxylin-stained nuclei and using them to train a slide-specific random-forest model. The trained model was then applied to images to generate a posterior map as an output. Each pixel value therefore represents the likelihood of that pixel belonging to a negative nucleus. Using the posterior map, we segmented the remaining negative nuclei [8]. We used indication-specific histological artifact detection, such as anthracotic pigment in lung tissue. For these and other reasons, such as differences in neoplastic growth patterns, customized IA scoring methods per indication were considered necessary. This enabled the generation of robust IA methods for CD8 parameters across all indications considered in this study. In the final step, we classified negative nuclei into fibroblast, lymphoid, and tumor nuclei. Again, prototypical nuclei were identified for each class based on the parameters per indication. Initially classified nuclei were used as input data to train an image-specific decision tree that was then used to classify all remaining nuclei. This strategy allowed us to account for some differences in the neoplastic growth patterns or other histological characteristics of the tumor immune response. Such additional characteristics are at present considered exploratory signatures, however, as they have not necessarily been validated to the degree that we validated CD8 IA.

**Measurement of CD8+ TILs in PD-L1–positive tumor**

In this study, we validated the IA enumeration of CD8+ TILs of the CD8/PD-L1 dual immunostain applied to tumor specimens from 24 NSCLC patients who did not meet screening criteria in clinical trial NCT01693562. To that end, we paired purple chromogen with CD8 and brown chromogen with PD-L1 (SP263 clone). Our standard CD8 IHC and the dual stain were applied to serial tumor sections. In our automatic classification assessment and validation approach, the cell annotations of two pathologists were applied to both single-stained and dual-stained images. For dual-stained images, CD8+ TILs were annotated as for other sample sets. The concordance values are shown in Additional file: Table S3. The CCC against consolidated annotations showed at least moderate agreement for the single stain (CCC = 0.81, CCC_lower = 0.77). For the dual stain, the criteria for moderate agreement were just missed (CCC = 0.73, CCC_lower = 0.63). Although the agreement with one of the two pathologists was substantial (CCC = 0.89, CCC_lower = 0.83), it was poor with the other pathologist (CCC = 0.66, CCC_lower = 0.46). We recognized that IA tended to detect more CD8+ TILs than the pathologist. In particular, IA better discriminated CD8+ TILs within intensely PD-L1–positive tumor nests (main text, Fig. 3) for which the color distinction was not convincing to the pathologist.

**References**

1. Althammer S, Steele K, Rebelatto M, et al. Combinatorial CD8+ and PD-L1+ cell densities correlate with response and improved survival in non-small cell lung cancer (NSCLC) patients treated with durvalumab. J Immunother Cancer*.* 2016;4(suppl 2):91.

2. Danaher P, Warren S, Dennis L, et al. Gene expression markers of tumor infiltrating leukocytes. J Immunother Cancer*.* 2017;5:18.

3. Yigitsoy M, Schmidt G. Hierarchical patch-based co-registration of differently stained histopathology slides. *Proc SPIE Int Soc Opt Eng.* 2017:1014009.

4. r Core Team. A language and environment for statistical computing. Available at [https://cran.r-project.org.](https://cran.r-project.org/web/packages/ggplot/index.html) Accessed May 5, 2017. 2016; A language and environment for statistical computing. Available at [https://cran.r-project.org.](https://cran.r-project.org/web/packages/ggplot/index.html) Accessed May 5, 2017. Available at: [https://cran.r-project.org.](https://cran.r-project.org) Accessed May 5, 2017.

5. Stevenson M, Nunes T, Heuer C, et al. epiR: tools for the analysis of epidemiological data. Available at [https://cran.r-project.org/web/packages/epiR/index.html.](https://cran.r-project.org/web/packages/epiR/index.html) Accessed May 5, 2017. 2017; epiR: tools for the analysis of epidemiological data. Available at [https://cran.r-project.org/web/packages/epiR/index.html.](https://cran.r-project.org/web/packages/epiR/index.html) Accessed May 5, 2017. Available at: [https://cran.r-project.org/web/packages/epiR/index.html.](https://cran.r-project.org/web/packages/epiR/index.html) Accessed May 5, 2017.

6. Therneau T, Grambsch PM. *Modeling Survival Data: Extending the Cox Model.* New York: Springer; 2000.

7. Wickham H. ggplot2: Elegant Graphics for Data Analysis. Available at [https://cran.r-project.org/web/packages/ggplot2/index.html.](https://cran.r-project.org/web/packages/ggplot2/index.html) Accessed May 5, 2017. 2009; ggplot2: Elegant Graphics for Data Analysis. Available at [https://cran.r-project.org/web/packages/ggplot/index.html.](https://cran.r-project.org/web/packages/ggplot/index.html) Accessed May 5, 2017. Available at: <https://cran.r-project.org/web/packages/ggplot2/index.html>.

8. Brieu N, Pauly O, Zimmermann J, Binnig G, Schmidt G, Slide-specific models for segmentation of differently stained digital histopathology whole slide images. Slide-specific models for segmentation of differently stained digital histopathology whole slide images. International Society for Optics and Photonics; February 27, 2016; San Diego, CA.
