# Supplementary material for: Measuring multiple parameters of CD8+ tumor-infiltrating lymphocytes in human cancers by image analysis
Source: J Immunother Cancer. 2018 Mar 6;6:20. doi: 10.1186/s40425-018-0326-x (PMC5839005; doi:10.1186/s40425-018-0326-x)
Supplement: Supplementary file 3 — Figure S1 IA classification of CD8+ lymphocytes. Figure S2 IA classification of elongate CD8+ lymphocytes. Figure S3 Elongate CD8+ TILS detected by IA are also CD3+. Figure S4 Tumor landscape of elongate CD8+ TILs. (DOCX 1795 kb) [file 40425_2018_326_MOESM3_ESM.docx]

**Measuring Multiple Parameters of CD8+ Tumor-Infiltrating Lymphocytes in Human Cancers by Image Analysis**

Steele KE, Tan TH, Korn R, Dacosta K, Brown C, Kuziora M, Zimmerman J, Laffin B, Widmaier M, Rognoni L, Cardenes R, Schneider K, Boutrin A, Martin P, Zha J, Wiester T

**Additional file 3: Supplementary Figures**

**Fig. S1.** IA classification of CD8+ lymphocytes. Low- (A) and high- (B) magnification views of CD8+ lymphocytes were classified (red) from a nonclinical excisional biopsy specimen of nonsquamous NSCLC. Shown are views of the corresponding unclassified digital images of CD8-immunostained tissue at low (C) and high (D) magnification. Comparison of the classified and unclassified images provides visual confirmation of the accuracy of detection of the IA scoring methods. Examination of the classified images further provides information about the density, distribution, and spatial localization of CD8+ lymphocytes in the tumor and surrounding resident lung tissue.


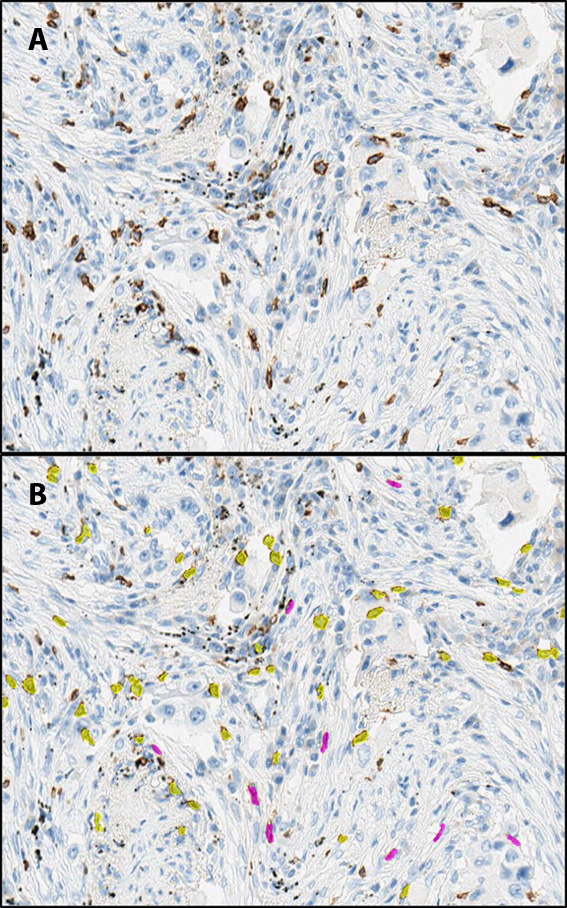


**Fig. S2.** IA classification of elongate CD8+ TILs. Shown are nonclassified (A) and classified (B) images of elongate CD8+ TILs in a non–small-cell lung carcinoma specimen. Specimen images were analyzed using a scoring method that discriminated between TILs with a length-to-width ratio of 2.3 or greater (elongate, highlighted red) and less than 2.3 (non-elongate, highlighted yellow). Visual inspection of IA results for multiple cases revealed that IA detected elongated CD8+ TILs if those cells were linearly oriented, as in these images. Because the pathologist could not visually apply the same length-to-width mathematical formula for multiple fields of view, we were not able to apply the automatic classification assessment and validation approach for this algorithm.


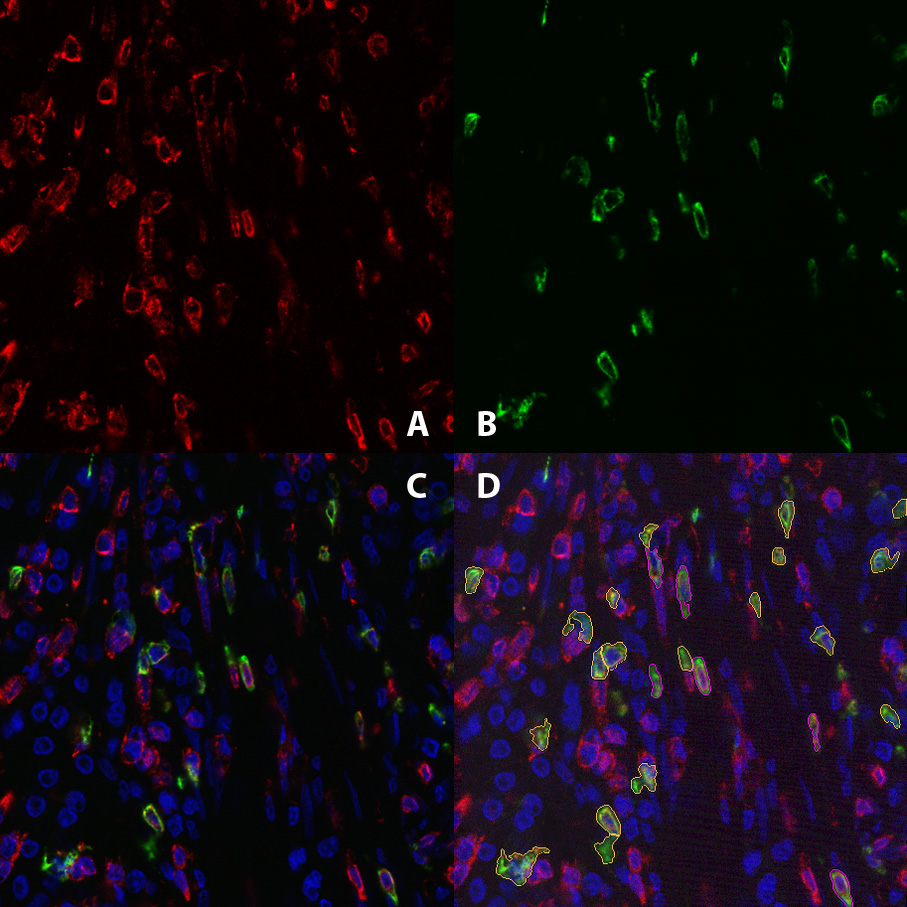


**Fig. S3.** Elongate CD8+ TILs detected by IA are also CD3+. Shown are separate immunofluorescence channels for CD3 (A, red) and CD8 (B, green) as well as combined channels (C, non-classified; and D, classified by IA). In the classified image, CD8+ TILs that met the scoring criteria as elongate (short arrows) are delineated with a red outline while non-elongate (long arrows) TILs are delineated with a yellow outline. All elongate CD8+ TILs thus classified exhibit at least some CD3 expression (A, short arrows) in this image. For this experiment, archived samples of NSCLC known to contain numerous elongate CD8+ TILS were immunolabeled using a dual immunofluorescence assay performed on the Ventana Discovery autostainer using antibodies to CD8 (clone SP239) and CD3 (SP162). The CD3 antibody was followed by incubation with anti-Rabbit OmniMap-HRP and the Discovery FITC kit (cat#760-232). The CD8 antibody was followed by incubation with anti-Rabbit OmniMap-HRP and the Discovery Cy5 kit. The samples were then stained with DAPI and coverslips were applied using ProLong Gold antifade media. Images of high magnification fields were acquired on a Leica SP5 Confocal microscope and saved as TIF files. CD3 antigen was arbitrarily represented as red and CD8 as green in these images. Images were analyzed in Definiens Developer and classified with the identical rule as applied to the CD8 single-labeled IHC samples where TILs with a length-to-width ratio of 2.3 or greater were classified as elongate (highlighted red) and less than 2.3 as non-elongate (highlighted yellow).

A

B

**Fig. S4.** Tumor landscape of elongate CD8+ TILs. Nonclinical specimens were analyzed to detect elongate and non-elongate TILs. Densities of elongate CD8+ TILs (A) were tabulated separately for TC and IM based on manual annotations of tumor regions. Individual specimen scores are plotted as a separate dot and connected to show the TC and IM scores for each one. For each tumor type, the Wilcoxon T values denote the degree of statistical difference between elongate CD8+ TIL densities in the TC versus the IM. Across these cancer types (B), we found that 10% or less of CD8+ TILs in both the IM and the TC were elongated in almost all cases. We are currently exploring this novel CD8 parameter in additional nonclinical and clinical tumor sets to better determine whether the numbers of elongate CD8+ TILs might have direct relevance to the immune response to cancer. GEC: gastroesophageal carcinoma; HNSCC: head and neck squamous-cell carcinoma; IM: invasive margin; LNSQ: nonsquamous non–small-cell lung carcinoma; PANC: pancreatic carcinoma; PROS: prostate carcinoma; RCC: renal cell carcinoma; TC: tumor center; TIL: tumor-infiltrating lymphocyte; UBC: urothelial bladder carcinoma.
